# *Salmonella*-superspreader hosts require gut regulatory T cells to maintain a disease-tolerant state

**DOI:** 10.1084/jem.20242431

**Published:** 2025-09-09

**Authors:** Blanda Di Luccia, Liliana M. Massis, Daniel S.C. Butler, Ramya Narasimhan, Sarah J. Ruddle, Trung H.M. Pham, José G. Vilches-Moure, Denise M. Monack

**Affiliations:** 1 Department of Microbiology and Immunology, Stanford University School of Medicine, Stanford, CA, USA; 2 Department of Cellular Microbiology, Max Planck Institute for Infection Biology, Berlin, Germany; 3 Department of Pediatrics, Stanford University School of Medicine, Stanford, CA, USA; 4 Department of Comparative Medicine, Stanford University School of Medicine, Stanford, CA, USA

## Abstract

Host–pathogen interactions involve two critical strategies: resistance, whereby hosts clear invading microbes, and tolerance, whereby hosts carry high pathogen burden asymptomatically. Here, we investigate mechanisms by which *Salmonella*-superspreader (SSP) hosts maintain an asymptomatic state during chronic infection. We found that regulatory T cells (Tregs) are essential for this disease-tolerant state, limiting intestinal immunopathology and enabling SSP hosts to thrive, while facilitating *Salmonella* transmission. Treg depletion in SSP mice resulted in decreased survival, heightened gut inflammation, and impairment of the intestinal barrier, without affecting *Salmonella* persistence. Colonic Tregs from SSP mice exhibited a unique transcriptomic profile characterized by the upregulation of type 1 inflammatory genes, including the transcription factor T-bet. In the absence of Tregs, we observed robust expansion of cytotoxic CD4^+^ T cells, with CD4^+^ T cell depletion restoring homeostasis. These results uncover a critical host strategy to establish disease tolerance during chronic enteric infection, providing novel insights into mucosal responses to persistent pathogens and chronic intestinal inflammation.

## Teaser

Di Luccia et al. demonstrate that gut regulatory T cells are crucial to maintain the disease-tolerant state of asymptomatic carriers during *Salmonella* chronic infection. These findings provide novel insights into mucosal responses to persistent pathogens.

## Graphical Abstract

**Figure:**
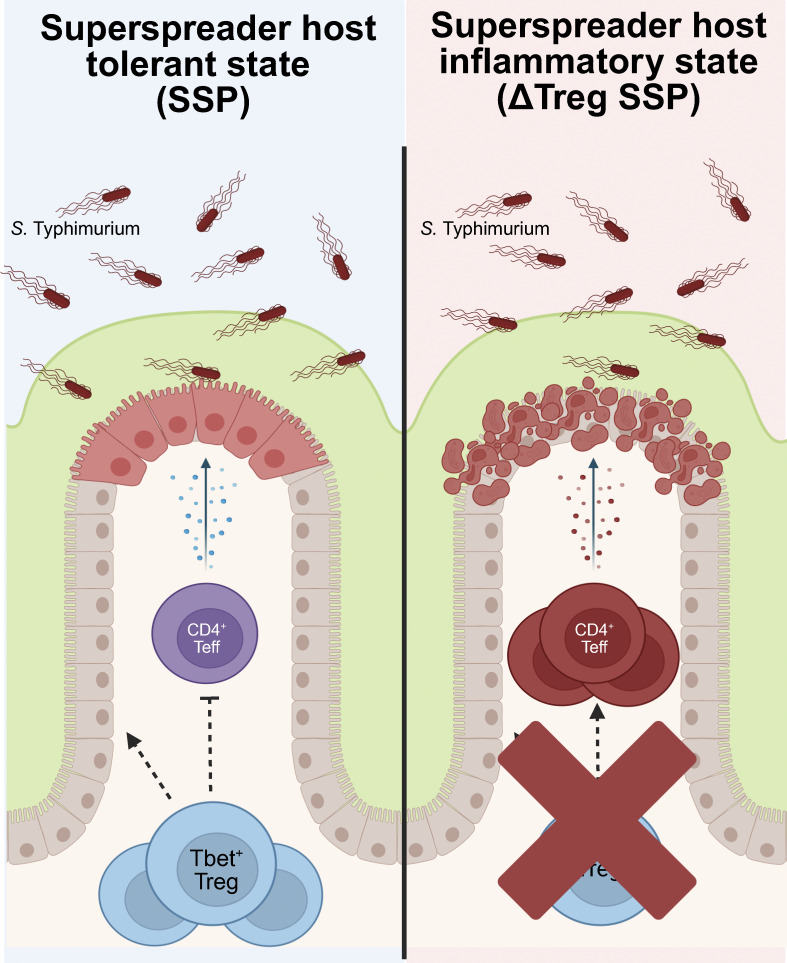

## Introduction

*Salmonella enterica* is an intracellular pathogen encompassing over 2,600 serovars, which can cause a variety of disease states in immunocompetent hosts, ranging from self-limiting gastroenteritis to life-threatening systemic illnesses. In addition to the severity of the pathology caused, a major concern is that certain *Salmonella* serovars can establish chronic, asymptomatic infections that enable efficient transmission of *Salmonella* between individuals. 1–4% of hosts infected with the human-restricted *Salmonella enterica* serovar Typhi become chronic carriers, continuing to shed the pathogen for up to a lifetime (Marzel et al., 2016; Parry et al., 2002). Although several studies have investigated the molecular mechanisms underlying pathogen persistence (Costerton et al., 1999; Helaine et al., 2014; Ruddle et al., 2023; Salama et al., 2013), additional work is needed to fully elucidate which host determinants regulate this asymptomatic state and ultimately how we can effectively leverage host mechanisms to better control pathogen transmission.

During host–pathogen interaction, the host can adopt defense mechanisms that result in either resistant or tolerant responses (Schneider and Ayres, 2008). Resistant hosts control infection and minimize disease-associated damage by reducing pathogen load. In contrast, tolerant hosts maintain long-term pathogen loads and develop responses that limit the pathogen’s impact on their health. Small subsets of carriers—often those with higher pathogen loads—can contribute disproportionately to disease spread. This phenomenon is described by the Pareto 80/20 rule, which states that 20% of carriers are responsible for 80% of disease transmission, potentially leading to fatalities in newly infected individuals (Woolhouse et al., 1997). Such carriers are referred to as “superspreaders.” Therefore, understanding the host factors that determine the asymptomatic state in superspreaders is crucial for mitigating the public health threat posed by uncontrolled disease transmission (Lloyd-Smith et al., 2005; Medzhitov et al., 2012; Paull et al., 2012).

Our group previously demonstrated that 129X1/SvJ mice can be chronically infected with *Salmonella enterica* serovar Typhimurium (*S*Tm) (Monack et al., 2004b). About 30% of these mice become superspreaders, shedding more than 10^8^ CFUs of *Salmonella* per gram of feces (Lawley et al., 2008). *S*Tm-infected superspreader (*Salmonella*-superspreader [SSP]) mice are considered tolerant hosts because they do not exhibit obvious signs of disease (e.g., ruffled fur, weight loss, diarrhea, or decrease in temperature) while shedding enough pathogen to infect naïve hosts (Lawley et al., 2008). These SSP mice possess a distinct splenic immune profile, characterized by neutrophil-dependent blunting of systemic T helper 1 (Th1) responses (Gopinath et al., 2013). Although *S*Tm infection is initially established at the intestinal barrier, very little is known about whether mucosal immune mechanisms support the host’s asymptomatic state.

In the gastrointestinal tract, the host’s immune system is continuously exposed to an enormous variety of benign antigens from commensal microbes and dietary molecules that can trigger unwanted inflammatory responses. Thus, tolerance mechanisms are critical for limiting damaging immune responses (Harrison and Powrie, 2013) and ensuring proper recognition of benign antigens at mucosal barriers. Regulatory T cells (Tregs) play a central role in establishing tolerance in tissues by dampening CD4^+^ and CD8^+^ T cell responses during inflammation (Dittmer et al., 2004; Suvas et al., 2003; Xu et al., 2018; Zhang et al., 2021). Moreover, Tregs can acquire the expression of transcription factors associated with distinct helper T cell subsets enabling them to suppress corresponding types of inflammatory responses in a context-specific manner (Oestreich and Weinmann, 2012). For instance, Rorγt^+^ Tregs encompass the majority of colonic Tregs that peripherally differentiate in response to antigens of commensal microbes (Lathrop et al., 2011), while Gata3^+^ Tregs (Schiering et al., 2014; Wohlfert et al., 2011) expand in response to IL-33 production upon tissue damage (Sefik et al., 2015).

Treg-mediated suppression has been shown to limit the immune response during intravenous *Salmonella* infection, resulting in an elevated pathogen load and persistence at systemic sites (Johanns et al., 2010). However, physiological transmission of *Salmonella* occurs via the fecal–oral route, and the interaction established by host immunity with the enteric pathogen at the intestinal barrier likely dictates the balance between resistance and tolerance. Understanding the role of intestinal Tregs during chronic *Salmonella* infection can provide insights into the establishment of asymptomatic carriers, and more broadly reveal strategies that facilitate pathogen persistence and transmission. Here, using a model of *Salmonella* chronic infection, we demonstrate that Tregs are critical for maintaining the disease-tolerant state of SSP hosts. Treg ablation during infection resulted in increased morbidity, loss of intestinal barrier integrity, and heightened inflammation, while pathogen burden remained unaffected. Our findings reveal that chronically infected superspreaders developed transcriptionally distinct populations of Tregs compared with uninfected mice. Colonic Tregs expressing T-bet accumulated during infection and suppressed the expansion of cytotoxic CD4^+^ T cells in superspreader hosts, thus ameliorating the intestinal immunopathology. Collectively, these results show that in SSP hosts an asymptomatic state is actively enforced by unique populations of Tregs, which sustain disease tolerance at the mucosal–pathogen interface.

## Results

### Asymptomatic SSP hosts develop a robust type 1 inflammatory response in the colon

**Figure S1.**
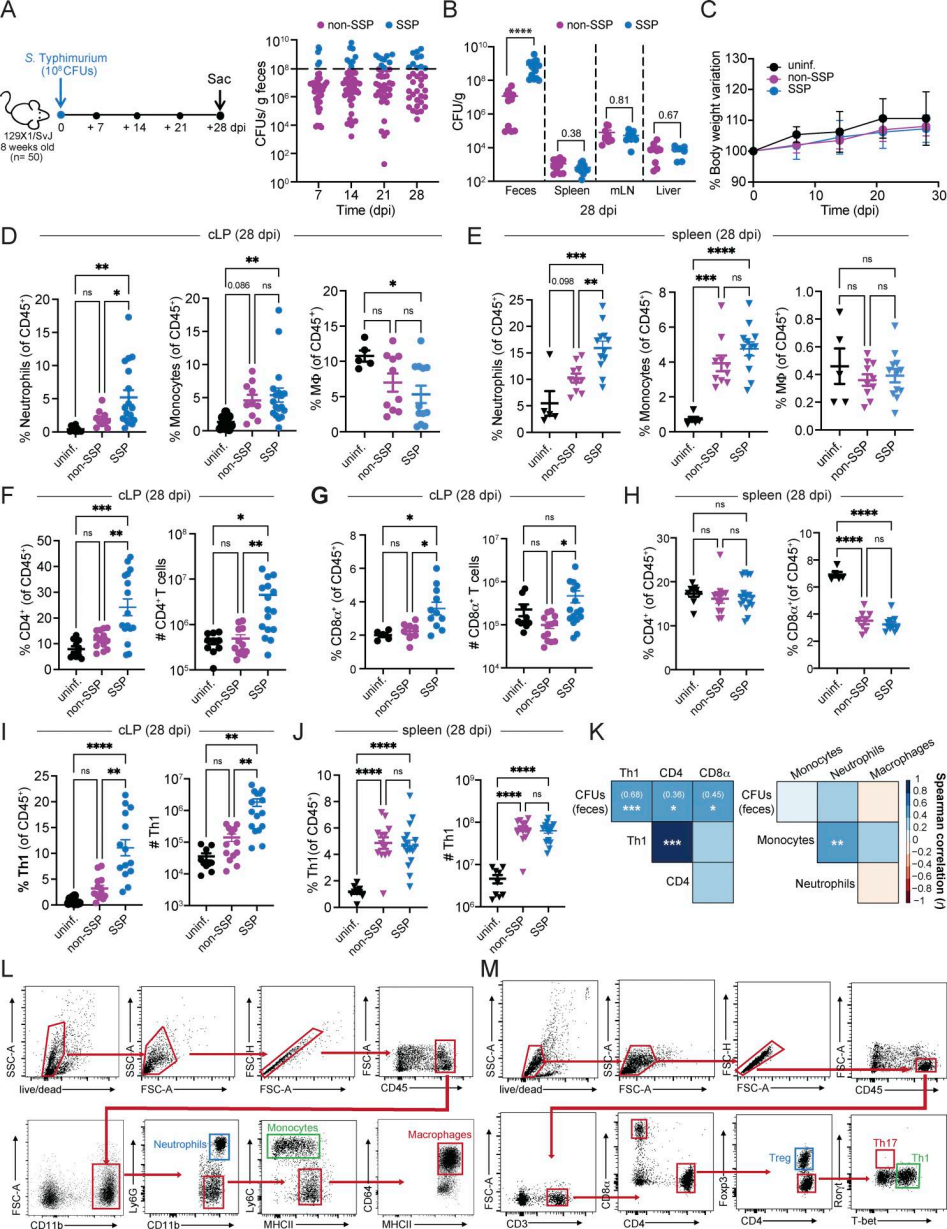
**Colonic and splenic immune responses of SSP and non-SSP mice at 28 dpi. (A)** Schematic representation of the experimental design. 129X1/SvJ mice (*n* = 10–50) were infected with *Salmonella* Typhimurium SL1344 (10^8^ CFUs; *S*Tm), and the fecal shedding (right) was monitored at the time indicated (7, 14, 21, 28 dpi). Each dot represents a single mouse; dark-pink circles indicate non-SSP mice, while light-blue circles indicate SSP mice. **(B)***S*Tm CFUs/g of feces, spleen, MLN, and liver collected from 129X1/SvJ at 28 dpi (*n* = 10–15). Dark-pink circles indicate non-SSP mice, and light-blue circles indicate SSP mice. **(C)** Percentage of body change variation of uninfected (black circle), SSP (light-blue circle), and non-SSP (dark-pink circle) mice at 7, 14, 21, 28 dpi (*n* = 10). **(D)** Frequency of neutrophils, monocytes, and macrophages (MΦ) isolated from the colonic lamina propria (cLP) of uninfected (black circle), non-SSP (dark-pink circle), and SSP (light-blue circle) mice at 28 dpi (*n* = 5–10). **(E)** Frequency of neutrophils, monocytes, and macrophages (MΦ) isolated from the spleen of uninfected (black triangle), non-SSP (dark-pink triangle), and SSP (light-blue triangle) mice at 28 dpi (*n* = 5–10). **(F and G)** Frequency and numbers of CD4^+^ T (F) and CD8α^+^ T (G) cells isolated from the colonic lamina propria of uninfected (black circle), non-SSP (dark-pink circle), and SSP (light-blue circle) mice at 28 dpi (*n* = 10–15). **(H)** Frequency of CD4^+^ and CD8α^+^ T cells isolated from the spleen of uninfected (black triangle), non-SSP (dark-pink triangle), and SSP (light-blue triangle) mice at 28 dpi (*n* = 10–15). **(I)** Frequency and numbers of Th1 cells isolated from the colonic lamina propria of uninfected (black circle), non-SSP (dark-pink circle), and SSP (light-blue circle) mice at 28 dpi (*n* = 10–15). **(J)** Frequency and numbers of Th1 cells isolated from the spleen of uninfected (black triangle), non-SSP (dark-pink triangle), and SSP (light-blue triangle) mice at 28 dpi (*n* = 10–15). **(K)** Spearman’s correlation matrix showing association between *Salmonella* fecal CFUs with Th1, CD4^+^, and CD8α^+^ T cells (right) or with neutrophils, monocytes, and macrophages (left), isolated from the colonic lamina propria of SSP and non-SSP hosts (*n* = 10–15). Spearman’s correlation coefficient (*r*) and relative P values (*P < 0.05, **P < 0.01, ***P < 0.001) are shown. **(L)** Gating strategy for flow cytometry used to identify neutrophils, monocytes, and macrophages in the colonic lamina propria and spleen of uninfected, non-SSP, and SSP mice. **(M)** Gating strategy for flow cytometry used to identify T cell subsets in the colonic lamina propria and spleen of uninfected, non-SSP, and SSP mice. Results are representative of at least two independent experiments and presented as means ± SEM. Normality was assessed by the D’Agostino–Pearson test. The Mann–Whitney U (B) test was used to compare two groups. One-way ANOVA (D–K) followed by post hoc Tukey’s test was performed for multiple groups comparisons (****P < 0.0001; ***P < 0.001; **P < 0.01; *P < 0.05; ns = not significant). MLN, mesenteric lymph node.

We previously developed a SSP mouse model, characterized by animals that shed high levels of *Salmonella* in their feces and can transmit disease to cohoused naïve hosts (Gopinath et al., 2013; Ruddle et al., 2023). In the current study, we investigated aspects of host tolerance during chronic infection using this model, specifically aiming to understand how hosts maintain a high bacterial burden of an infectious pathogen while remaining asymptomatic (no obvious weight loss or diarrhea). 129X1/SvJ mice were orally infected with 10^8^ CFUs of *S*Tm SL1344, and the fecal bacterial load at 7, 14, 21, and 28 days post-infection (dpi) was used to classify mice in two groups: SSP (natural SSP; >10^8^ CFU *Salmonella*/g feces) and non-SSP (<10^8^ CFU *Salmonella*/g feces) hosts (Fig. S1 A). Since SSP mice and non-SSP mice both carry *S*Tm at 28 dpi (Fig. S1 B), we compared them with each other to highlight unique changes in the immune response driven by higher bacterial burden and with uninfected mice to identify changes triggered by the infection itself.

During infection, both SSP and non-SSP mice showed no weight loss and even exhibited weight gain similar to uninfected animals (Fig. S1 C). Additionally, SSP and non-SSP animals had similar levels of *Salmonella* CFUs in systemic organs (spleen, liver, mesenteric lymph nodes) at 28 dpi, despite differences in fecal *Salmonella* burden (Fig. S1 B). The high *Salmonella* burden in the feces of SSP mice, combined with the lack of visible signs of pathology, led us to hypothesize that the colonic immune response of SSP mice was a key host determinant in the development of the asymptomatic state. We next analyzed the host immune response in the colonic lamina propria of SSP animals, comparing them with non-SSP and uninfected controls. Additionally, we analyzed splenic immune cells at 28 dpi to differentiate between systemic responses and those specific to the mucosal barrier.

SSP mice showed greater frequencies of neutrophils and monocytes in the colonic lamina propria and spleen (Fig. S1, D, E, and L) compared with their uninfected counterparts, consistent with our previous findings at systemic sites (Gopinath et al., 2013; Lawley et al., 2008). Additionally, SSP mice showed a decreased frequency of resident macrophages compared with the uninfected controls, likely due to the increased presence of *Salmonella* virulence factors that cause macrophage death and facilitate pathogen escape from phagosomes (Hernandez et al., 2003; Monack et al., 2001). In contrast, non-SSP hosts displayed no significant changes in the frequencies of colonic neutrophils, monocytes, and macrophages compared with uninfected mice (Fig. S1 D), although they did show increased levels of splenic monocytes similar to those observed in SSP mice (Fig. S1 E). Notably, SSP mice had greater abundance of CD4^+^ and CD8α^+^ T cells (Fig. S1, F, G, and M) in the colonic lamina propria, but not in the spleen (Fig. S1 H), highlighting the distinct characteristics of mucosal immune responses compared with systemic sites. Additionally, colonic Th1 cells (T-bet^+^ CD4^+^ T cells) (Fig. S1 I) were increased only in SSP hosts, while splenic Th1 cells were elevated in both SSP and non-SSP hosts (Fig. S1 J). Overall, only the increased frequency of Th1, CD4^+^, and CD8α^+^ T cells in the colonic lamina propria of both SSP and non-SSP mice showed a positive correlation with the fecal shedding levels, as indicated by Spearman’s correlation coefficients (Fig. S1 K). These findings demonstrate that SSP hosts develop a distinct colonic myeloid and T cell response compared with non-SSP and uninfected animals, and that the colonic response is distinct from the splenic immune response.

**Figure 1.**
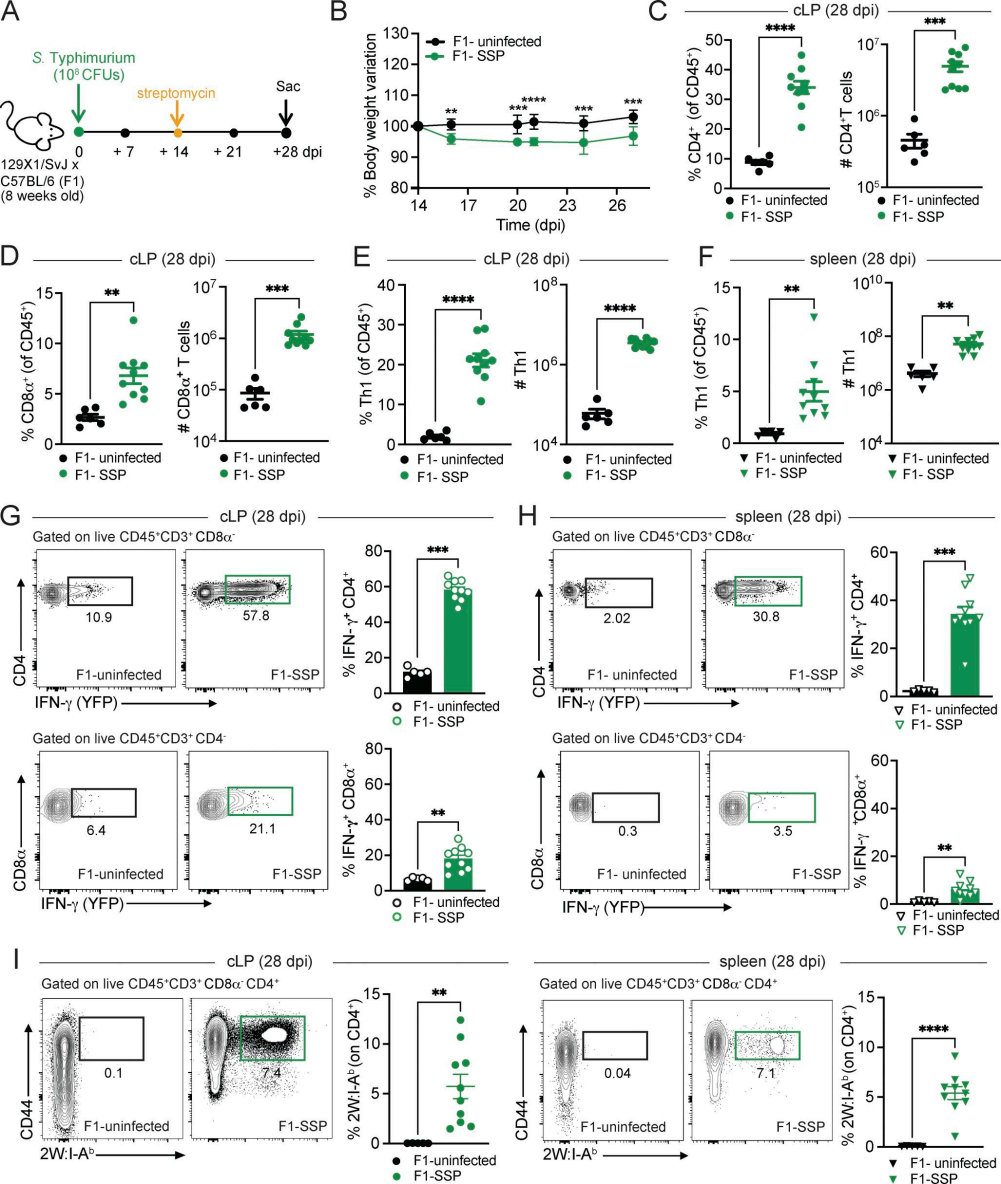
**Asymptomatic SSP mice show a robust type 1 inflammatory response in the colon at 28 dpi. (A)** Schematic representation of the experimental design. F1 mice (129X1/SvJ × C57BL/6; *n* = 10) were infected with *Salmonella* Typhimurium SL1344 (10^8^ CFUs; *S*Tm) and treated with streptomycin (5 mg) at 14 dpi to induce the SSP state. **(B)** Percentage of body change variation of F1-uninfected (black circle) and F1-SSP (green circle) mice after streptomycin treatment at 14 dpi (*n*= 10). **(C and D)** Frequency and numbers of CD4^+^ and CD8α^+^ T cells isolated from the colonic lamina propria (cLP) of F1-uninfected (black circle) and F1-SSP (green circle) mice at 28 dpi (*n* = 5–10). **(E)** Frequency and numbers of Th1 cells isolated from the colonic lamina propria of uninfected (black circle) and SSP (green circle) mice at 28 dpi (*n* = 5–10). **(F)** Frequency and numbers of Th1 cells isolated from the spleen of uninfected (black triangle) and SSP (green triangle) mice at 28 dpi (*n* = 5–10). **(G)** Representative FACS plots and frequency of IFN-γ–producing CD4^+^ T cells (top) and CD8α^+^ T cells (bottom) isolated from the colonic lamina propria of F1-uninfected and F1-SSP mice at 28 dpi (*n* = 5–10). **(H)** Representative FACS plots and frequency of IFN-γ–producing CD4^+^ T cells (top) and CD8α^+^ T cells (bottom) isolated from the spleen of F1-uninfected and F1-SSP mice at 28 dpi (*n* = 5–10). **(I)** Representative FACS plots and frequency of tetramer^+^ (2W:I-A^b^) CD4^+^ T cells isolated from the colonic lamina propria (circle) and spleen (triangle) of F1-uninfected and F1-SSP mice at 28 dpi (*n* = 5–10). Results are representative of at least two independent experiments and presented as means ± SEM. Normality was assessed by the D’Agostino–Pearson test. Two-way ANOVA (A) followed by post hoc Sidak’s test was performed for multiple comparisons. The Mann–Whitney U (C–I) test was used to compare two groups (****P < 0.0001; ***P < 0.001; **P < 0.01; ns = not significant). See also Figs. S1 and S2.

**Figure S2.**
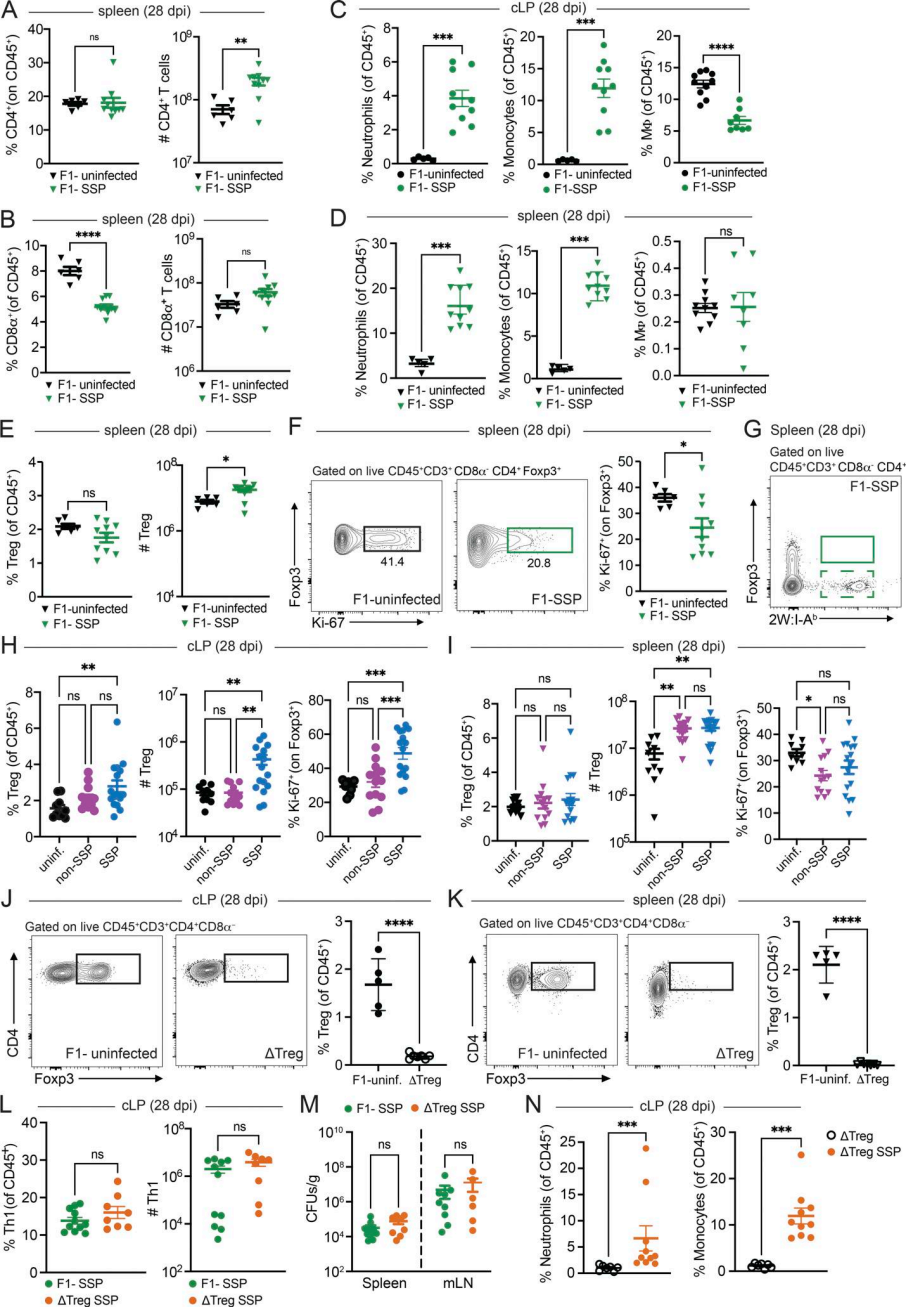
**Colonic and splenic immune responses of F1-SSP and ΔTreg SSP mice at 28 dpi.** **(A and B)** Frequency and numbers of CD4^+^ T (A) and CD8α^+^ T (B) cells isolated from the spleen of F1-uninfected (black triangle) and F1-SSP (green triangle) mice at 28 dpi (*n* = 10–15). **(C)** Frequency of neutrophils, monocytes, and macrophages (MΦ) isolated from the colonic lamina propria of F1-uninfected (black circle) and F1-SSP (green circle) mice at 28 dpi (*n* = 5–10). **(D)** Frequency of neutrophils, monocytes, and macrophages (MΦ) isolated from the spleen of F1-uninfected (black triangle) and F1-SSP (green triangle) mice at 28 dpi (*n* = 5–10). **(E)** Frequency and numbers of Tregs isolated from the spleen of F1-uninfected (black triangle) and F1-SSP (green triangle) mice at 28 dpi (*n* = 10–15). **(F)** Representative FACS plots and frequency of Ki-67^+^ Tregs isolated from the spleen of F1-uninfected (black triangle) and F1-SSP (green triangle) mice at 28 dpi (*n* = 10–15). **(G)** Representative FACS plot of tetramer^+^ (2W:I-A^b^) Foxp3^+^ CD4^+^ T cells isolated from the spleen of F1-SSP mice at 28 dpi. **(H)** Frequency and numbers of Tregs (left) and Ki-67^+^ Tregs (right) isolated from the colonic lamina propria of uninfected (black circle), non-SSP (dark-pink circle), and SSP (light-blue circle) mice at 28 dpi (*n* = 10–15). **(I)** Frequency and numbers of Tregs (left) and Ki-67^+^ Tregs (right) isolated from the spleen of uninfected (black triangle), non-SSP (dark-pink triangle), and SSP (light-blue triangle) mice at 28 dpi (*n* = 10–15). **(J and K)** Representative FACS plots and frequency of Tregs in the colonic lamina propria (J) and spleen (K) of uninfected F1-Foxp3^DTR^ (F1-uninfected) and ΔTreg after DT treatment, at 28 dpi (*n* = 5). **(L)** Frequency and numbers of Th1 cells detected in the colonic lamina propria of F1-SSP (green circle) and ΔTreg SSP (orange circle) at 28 dpi (*n* = 8–10). **(M)***S*Tm CFU counts in the spleen and MLNs of F1-SSP (green triangle) and ΔTreg SSP (orange triangle) mice at 28 dpi (*n* = 6–10). **(N)** Frequency of neutrophils (left panel) and monocytes (right panel) isolated from the colonic lamina propria of uninfected ΔTreg (open circle) and ΔTreg SSP (orange circle) mice at 28 dpi (*n* = 8–10). Results are representative of at least two independent experiments and presented as means ± SEM. Normality was assessed by the D’Agostino–Pearson test. The Mann–Whitney U (A–F and J–N) test was used to compare two groups (****P < 0.0001; ***P < 0.001; **P < 0.01; *P < 0.05; ns = not significant). One-way ANOVA (H and I) followed by post hoc Tukey’s test was performed for multiple groups comparisons. MLN, mesenteric lymph node.

To gain mechanistic insights into the SSP mucosal T cell responses, we next transitioned to a streptomycin-induced SSP mouse model, previously described by our group (Gopinath et al., 2013; Lawley et al., 2008). This model allowed us to utilize the *in vivo* genetic tools available on the C57BL/6 background, while also overcoming the *Nramp1*-dependent susceptibility of C57BL/6 mice (Monack et al., 2004a). Briefly, 129X1/SvJ mice were crossed with C57BL/6 mice; the F1 progeny were orally infected with *S*Tm (Fig. 1 A), and 2 wk later received a one-time treatment with streptomycin to overcome microbiota-mediated colonization resistance (Jacobson et al., 2018) and to induce the SSP state. Notably, upon this treatment all mice become SSP compared with ∼ 30% SSP observed in the natural SSP model, enhancing the penetrance of the superspreader phenotype and allowing for more consistent analysis. These induced SSP (F1-SSP) mice exhibited a modest weight loss (2–4% body weight variation) only after streptomycin treatment and then maintained a stable weight gain in the following weeks of infection (Fig. 1 B), resembling the natural SSP state (Fig. S1 C). Additionally, F1-SSP immune responses phenocopied those observed in the natural SSP mice at 4 wk after infection. Specifically, F1-SSP mice showed increased CD4^+^ and CD8α^+^ T cells (Fig. 1, C and D) in the colonic lamina propria, while in the spleen, only CD4^+^ T cells increased (Fig. S2, A and B). They showed an enhanced frequency of neutrophils and monocytes, but not macrophages, in the colonic lamina propria and spleen (Fig. S2, C and D). Moreover, F1-SSP mice exhibited increased Th1 in both mucosal (Fig. 1 E) and systemic tissues (Fig. 1 F) demonstrating the presence of a sustained type 1 inflammatory response similar to what is observed in the natural SSP hosts.

Next, we sought to determine whether the enhanced T cell populations were still functionally active 4 wks after infection. We utilized interferon-γ (IFN-γ) reporter mice (GREAT mice) to detect cytokine production by these cells in vivo. 129X1/SvJ mice were crossed with GREAT-C57BL/6 reporter mice (Reinhardt et al., 2009) to generate F1-GREAT mice, which were infected with *S*Tm and treated with streptomycin to establish the SSP state. We observed that CD4^+^ T cells were producing robust amounts of IFN-γ in the colon and spleen (Fig. 1, G and H) of SSP hosts, while CD8α^+^ T cells also produced IFN-γ at both sites, although to a lesser extent (Fig. 1, G and H). We next asked whether colonic *Salmonella*-specific CD4^+^ T cells were still present at 4 wk after infection. To examine this, F1 mice were infected with *S*Tm-2W1S, a strain of *Salmonella* Typhimurium SL1344 carrying the sequence of a specific immunogenic peptide (2W1S_52–68_), and treated with streptomycin to induce the SSP state. CD4^+^ T cells specific for the 2W1S_52-68_ peptide were identified by tetramer staining, and we observed a large population tetramer^+^ cell (2W:I-A^b^) in the colon of infected mice, which were similarly abundant in the spleen (Fig. 1 I). All tetramer^+^ cells were also positive for CD44, consistent with an activated phenotype and an ongoing immune response to persistent pathogen antigens. These data collectively demonstrate that SSP hosts harbor a sustained and active type 1 inflammatory response in the colon at 4 wk after infection, despite appearing asymptomatic and lacking obvious clinical symptoms of colitis such as weight loss or diarrhea.

### Tregs are crucial for maintaining intestinal tolerance in SSP hosts

**Figure 2.**
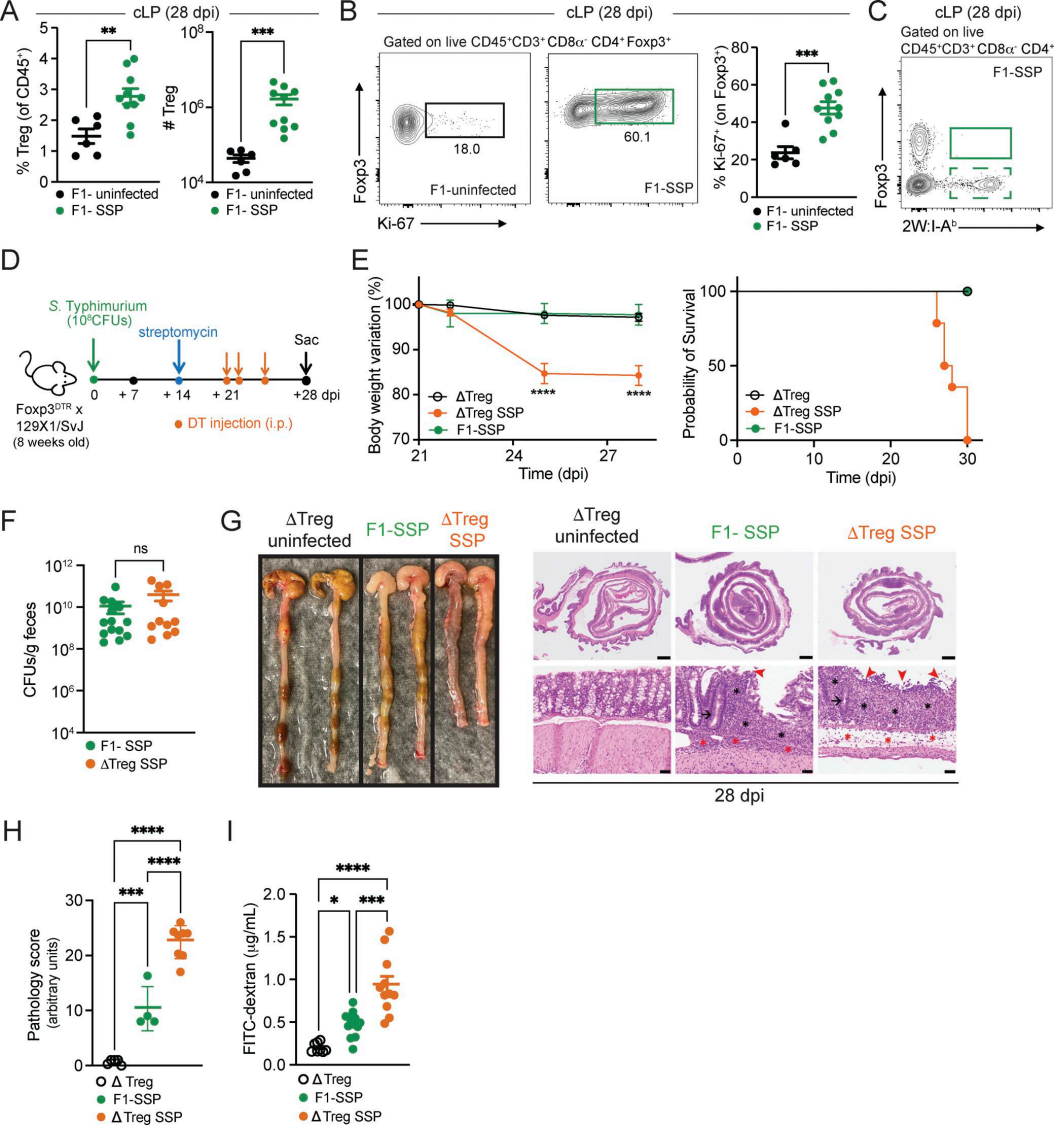
**Gut Tregs control intestinal immune pathology in SSP hosts. (A)** Frequency and numbers of Tregs isolated from the colonic lamina propria (cLP; circle) of F1-uninfected and F1-SSP mice at 28 dpi (*n* = 5–10). **(B)** Representative FACS plots and frequency of Ki-67^+^ Tregs isolated from the colonic lamina propria of F1-uninfected and F1-SSP mice at 28 dpi (*n* = 5–10). **(C)** Representative FACS plot of tetramer^+^ (2W:I-A^b^) Foxp3^+^ CD4^+^ T cells isolated from the colonic lamina propria of F1-SSP mice at 28 dpi. **(D)** Schematic representation of the experimental design. F1-Foxp3^DTR^ mice (*n* = 10) were infected or not with *Salmonella* Typhimurium SL1344 (10^8^ CFUs; *S*Tm), treated with streptomycin to induce SSP at 14 dpi, and injected (i.p.) or not with DT to deplete Tregs (ΔTreg) at 21, 22, and 25 dpi, as indicated. **(E)** Percentage of body weight variation and survival curve of ΔTreg (open black circle), ΔTreg superspreader (ΔTreg SSP; orange circle), and F1-SSP (green circle) mice (*n* = 10–15). **(F)***S*Tm CFUs measured in the feces of F1-SSP and ΔTreg SSP at 28 dpi (*n* = 10–15). **(G)** Representative images (left) and H&E staining (right) of colonic tissues of ΔTreg, F1-SSP, and ΔTreg SSP at 28 dpi, as indicated. Magnification of H&E sections: upper panels = 1.25×; lower panels = 20×. Scale bars: upper panels = 1.0 mm; lower panels = 50 μm. Inflammation in the lamina propria (black asterisks) and in the submucosa (red asterisk); goblet cells (black arrows); edema in the submucosa (red asterisks); areas of ulceration (red arrowheads). **(H)** Pathology scores assigned to colonic tissue sections of ΔTreg, F1-SSP, and ΔTreg SSP at 28 dpi (*n* = 4–8). **(I)** FITC-dextran detected in the serum of ΔTreg, F1-SSP, and ΔTreg SSP mice at 28 dpi (*n* = 5–10). Results are representative of at least two independent experiments and presented as means ± SEM. Normality was assessed by the D’Agostino–Pearson test. The Mann–Whitney U test (A, B, and F) was used to compare two groups. One-way ANOVA (H and I) followed by post hoc Tukey’s test or Two-way ANOVA (E) followed by post hoc Sidak’s test was performed for multiple comparisons (****P < 0.0001; ***P < 0.001; **P < 0.01; *P < 0.05; ns = not significant). See also Fig. S2 and Table S1.

Given that SSP mice exhibit an asymptomatic state despite a robust colonic Th1 response, we hypothesized that this inflammatory response was counterbalanced by a tolerogenic response at the intestinal barrier. Previous studies have highlighted the crucial role of Tregs in modulating immune responses during infections and in maintaining gut homeostasis (Clay et al., 2020; Harrison and Powrie, 2013). Therefore, we explored the role of Tregs in the SSP model, investigating their impact on pathogen burden and tissue homeostasis. Tregs (Foxp3^+^CD4^+^ T cells) were significantly increased in frequency and numbers in the colonic lamina propria of F1-SSP mice, compared with uninfected controls at 28 dpi (Fig. 2 A).

Intestinal Tregs from F1-SSP mice were also highly proliferative, with heightened expression of Ki-67, compared with uninfected hosts (Fig. 2 B). Notably, the enhanced proliferation of Tregs was only observed in the colon of F1-SSP hosts, whereas splenic Treg proliferation was similar between F1-SSP and uninfected mice (Fig. S2, E and F). We next asked whether the enhanced proliferation of colonic Tregs was due to the expansion of *Salmonella*-specific Tregs, and utilized the *S*Tm-2W1S strain to infect F1 mice and detect 2W1S-specific Tregs. Unexpectedly, we observed that no Tregs exhibited specificity to the *Salmonella*-antigen 2W1S_52-68_ in the colon or spleen (Fig. 2 C and Fig. S2 G), possibly because they are specific for different *Salmonella* antigens or because they are non–*Salmonella*-specific Tregs. Finally, a similar Treg phenotype was observed in natural SSP mice. Colonic Tregs were more abundant and highly proliferative (Fig. S2 H) compared with non-SSP and uninfected mice, while splenic Tregs (Fig. S2 I) were modestly increased in cell numbers and Ki-67 expression was similar to non-SSP and uninfected controls.

To determine the impact of Tregs during chronic infection, we utilized a genetic mouse model in which only Tregs express the diphtheria toxin receptor (DTR), allowing for the specific ablation of these cells in SSP mice during infection by systemic administration of diphtheria toxin (DT). Foxp3^DTR^ mice (Kim et al., 2007) on the C57BL/6 background were crossed with 129X1/SvJ male mice to generate F1-Foxp3^DTR^ mice, which were infected with *S*Tm and treated with streptomycin to induce the SSP state (F1-SSP; Fig. 2 D). Tregs were depleted in F1-SSP by DT injection (ΔTreg SSP) starting at 21 dpi, and the Treg ablation was confirmed by the absence of Foxp3^+^ T cells in the gut and spleen of ΔTreg mice (Fig. S2, J and K). ΔTreg SSP mice exhibited rapid weight loss and decreased survival, whereas uninfected ΔTreg mice and F1-SSP did not show any weight loss for the duration of the experiment (Fig. 2 E). Surprisingly, the ablation of Tregs in F1-SSP mice (ΔTreg SSP) did not impact the total abundance of colonic Th1 cells (Fig. S2 L), nor did it alter the pathogen load in the gut or systemic organs, compared with Treg-sufficient F1-SSP hosts (Fig. 2 F and Fig. S2 M).

Moreover, ΔTreg SSP mice presented with a severe colonic pathological score, assessed by inflammation in the lamina propria (black asterisks) and submucosa (red asterisks), ulceration (red arrowheads), paucity of goblet cells (black arrow), and edema (red asterisks) (Fig. 2, G and H), as well as with an increased colonic infiltration of neutrophils and monocytes (Fig. S2 N). Uninfected ΔTreg mice had a normal pathological score and minimal colonic myeloid cell infiltration at the end of the treatment.

Given the striking morbidity and enhanced colonic inflammatory state of ΔTreg SSP, we hypothesized that the integrity of the intestinal epithelial barrier was compromised. To test this, we measured the permeability of the gut epithelial barrier of ΔTreg SSP and F1-SSP mice using the fluorescein isothiocyanate–dextran (FITC-dextran) assay. Mice were orally gavaged with FITC-dextran at 28 dpi, and the accumulation of this compound was measured in the serum 4 h after administration. Indeed, ΔTreg SSP mice exhibited significantly greater intestinal barrier permeability compared with ΔTreg uninfected controls and F1-SSP mice (Fig. 2 I), in line with the enhanced colonic epithelial damage observed by histological examination. Altogether, these findings indicate that during chronic *Salmonella* infection, gut Tregs are essential for preserving tissue integrity and maintaining the asymptomatic state of the host, but do not directly impact pathogen burden.

### Colonic Tregs exhibit a distinct transcriptomic profile upon *Salmonella* infection

**Figure S3.**
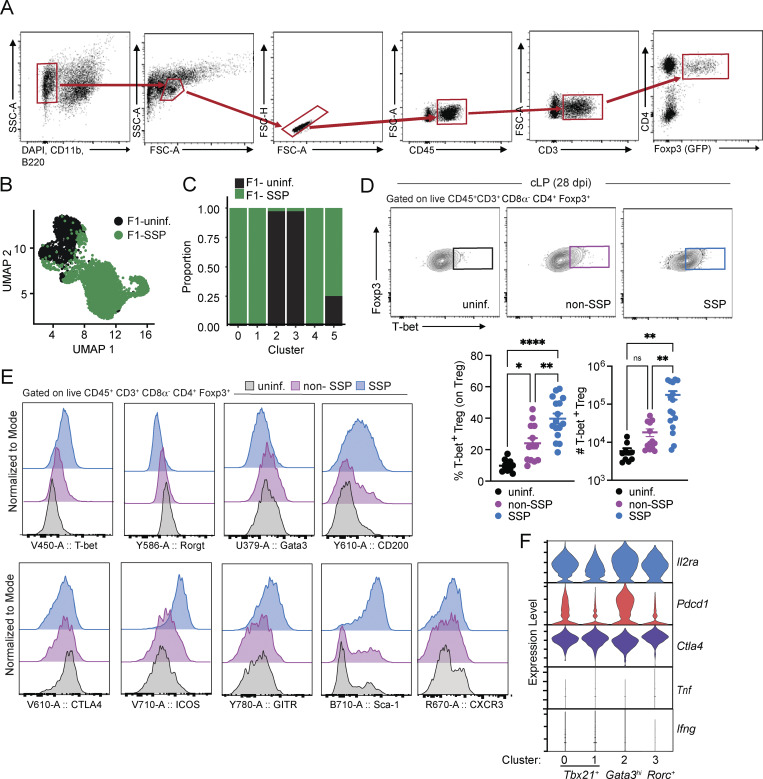
**Additional data related to the characterization of colonic Tregs isolated from F1-SSP mice and from natural SSP compared with non-SSP mice at 28 dpi. (A)** Gating strategy used for sorting Tregs from colonic lamina propria of F1-uninfected and F1-SSP mice at 28 dpi. **(B)** Uniform Manifold Approximation and Projection (UMAP) plot showing the clustering of colonic Treg based on condition and combination (uninfected/SSP). **(C)** Barplot showing the contribution of each condition to the different Treg clusters. **(D)** Representative FACS plots, frequency, and numbers of T-bet^+^ Tregs isolated from the colonic lamina propria (cLP) of uninfected (black circle), non-SSP (dark-pink circle), and SSP (light-blue circle) mice at 28 dpi (*n* = 5–10). Results are representative of at least two independent experiments and presented as means ± SEM. One-way ANOVA followed by post hoc Tukey’s test was performed for multiple groups comparisons (****P < 0.0001; **P < 0.01; *P < 0.05; ns = not significant). **(E)** Representative flow cytometry histograms showing expression levels of the indicated markers in colonic Tregs isolated from of uninfected (black line), non-SSP (dark-pink line), and SSP (light-blue line) mice at 28 dpi. **(F)** Violin plots showing the expression levels of *Il2ra*, *Pdcd1*, *Ctla4*, *Tnf*, and *Ifng* among clusters 0 and 1, present in F1-SSP mice, versus clusters 2 and 3, mostly represented in uninfected F1 mice.

**Figure 3.**
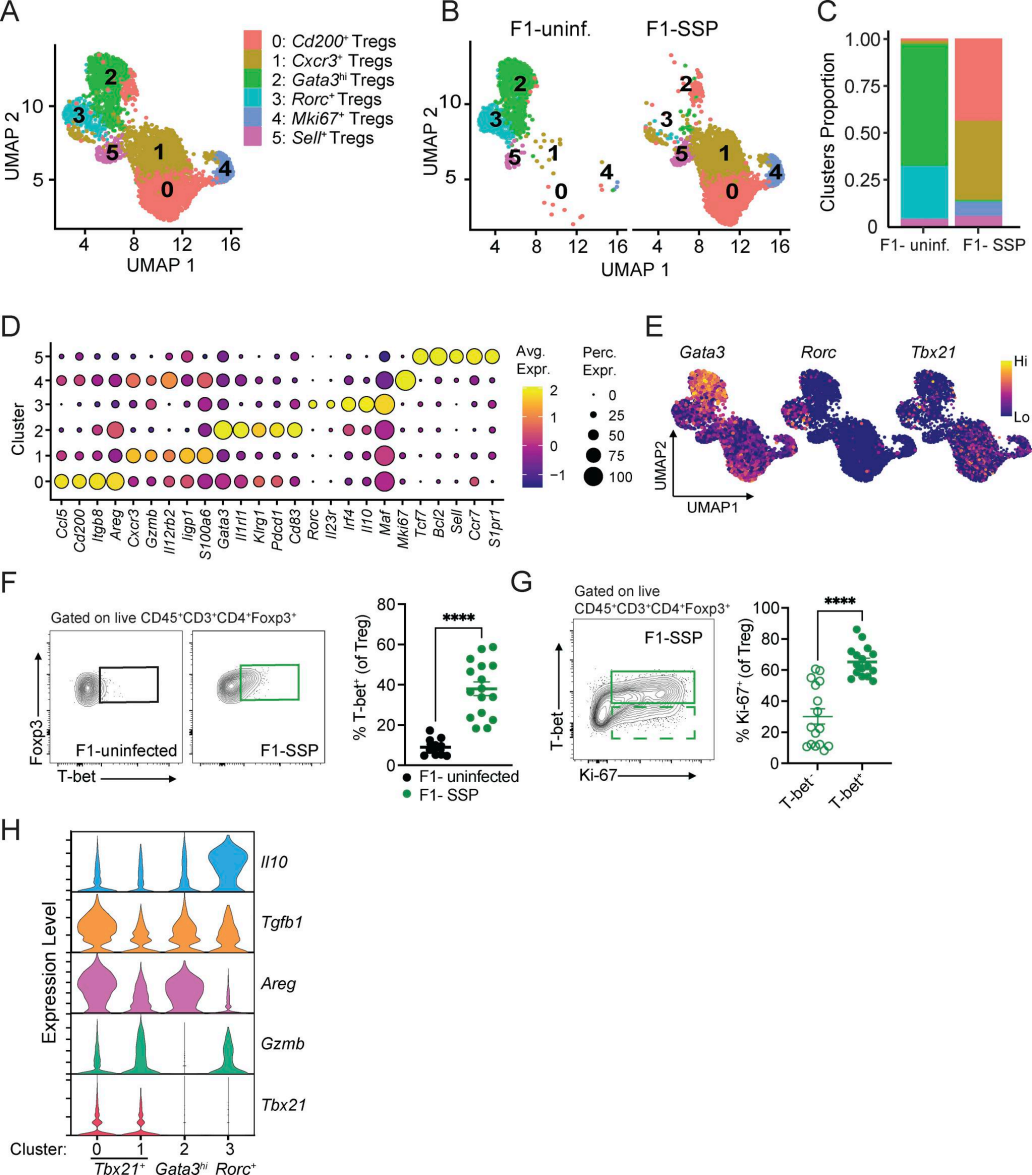
**scRNA-seq of colonic SSP Tregs reveals their distinct profile compared with Tregs from uninfected hosts. (A)** UMAP plot of colonic Treg clusters (0–5) generated by merging uninfected and SSP conditions. **(B)** UMAP plot of colonic Treg clusters (0–5) represented in each condition separately (uninfected/SSP). **(C)** Barplot showing the proportion of each Treg cluster of F1-uninfected or F1-SSP mice. **(D)** Dot plot representing a list of genes manually curated to identify the different gene expression signatures defining each cluster. The size of the dot represents the percentage of the cells in each cluster expressing a specific marker, while the color is a measure of the average expression of that gene in a specific cluster. **(E)** UMAP plots showing the expression of *Gata3*, *Rorc*, and *Tbx21* among all the identified Treg clusters. **(F and G)** Representative FACS plot and frequency of T-bet^+^ Tregs (F) and Ki-67^+^ T-bet^+^ Tregs (G) detected in the colonic lamina propria of F1-uninfected or F1-SSP mice (*n* = 5–10). Results are representative of two independent experiments and presented as means ± SEM. Normality was assessed by the D’Agostino–Pearson test. The Mann–Whitney U test was used to compare two groups (****P < 0.0001). **(H)** Violin plots showing the expression levels of *Il10*, *Tgfb1*, *Areg*, *Gzmb*, and *Tbx21* among clusters 0 and 1, only present in F1-SSP mice, versus clusters 2 and 3, only represented in uninfected F1 mice. See also Fig. S3.

Treg cells are a heterogeneous population defined not only by their tissue localization but also by the nature of the stimuli they encounter (Campbell and Koch, 2011; Gopinath et al., 2012). To gain deeper insights into the specific subtypes of Tregs inhabiting the colonic lamina propria of SSP hosts, we sorted colonic Foxp3-GFP^+^ Tregs from infected (F1-SSP) and uninfected (F1-uninf.) reporter mice (Fig. S3 A) and performed single-cell RNA sequencing (scRNA-seq). After quality control measures, we identified 5,732 cells assigned to six clusters (Fig. 3 A), manually defined based on population markers reported in previous studies (Miragaia et al., 2019; Trujillo-Ochoa et al., 2023). Examination of these clusters from SSP and uninfected mice revealed that clusters 2 and 3 were mostly present in uninfected hosts, while clusters 0, 1, and 4 were unique to SSP hosts (Fig. 3, B and C; and Fig. S3, B and C). Finally, cluster 5 was shared between colonic Tregs from SSP and uninfected mice.

In line with previous studies (Miragaia et al., 2019; Schiering et al., 2014), uninfected mice harbored a population of Tregs with a non-lymphoid tissue gene signature, resembling thymic-derived cells (cluster 2: *Gata3*^*+*^ Tregs) (Fig. 3 D). This subset is distinguished by the expression of *Gata3*, *Il1rl1*, *Klrg1*, *Pdcd1*, *Areg*, and *Cd83* genes. We also identified Tregs with a suppressive gene signature (cluster 3: *Rorc*^*+*^ Tregs) that resembled peripherally derived cells (Ohnmacht et al., 2015; Sefik et al., 2015), expressing genes like *Rorc*, *Irf4*, *Il10*, and *Il23r*. Furthermore, a population of Tregs expressing a lymphoid tissue-like gene signature was observed (cluster 5: *Sell*^*+*^ Tregs), characterized by the expression of genes like *Sell*, *Ccr7*, *S1pr1*, and *Tcf7*, and likely consisted of cells that had migrated to the gut from a different site (Fig. 3 D). In SSP hosts, we identified a subset of Tregs that expressed *Ccl5*, *Cd200*, *Itgb8*, and *Areg* (cluster 0: *Cd200*^*+*^ Treg) and *Cxcr3*, *Gzmb*, *Il12rb2*, and *ligp1* (cluster 1: *Cxcr3*^*+*^ Treg) (Fig. 3 D). Most of the genes that define clusters 0 and 1 are known to be induced by IFN-γ and likely reflect the type 1 inflammatory microenvironment where they reside. Lastly, cluster 4 (*Mki67*^*+*^ Tregs) Tregs exhibited the high expression of genes associated with cell proliferation, such as *Mki67*, *Hist1h1b*, and *Hist1h2ae*.

Tregs are known to upregulate transcription factors typically associated with helper T cell subsets in response to different microenvironments. We observed that colonic Tregs in SSP mice (clusters 0, 1, and 4) upregulated T-bet (*Tbx21*), the canonical Th1 transcription factor, which was minimally present in uninfected mice (clusters 2 and 3; Fig. 3 E). We validated that colonic Tregs from SSP mice expressed T-bet protein (Fig. 3 F) and observed that T-bet^+^ Tregs were more proliferative than the T-bet^−^ Tregs (Fig. 3 G). Similar to F1-SSP mice, increased abundance of T-bet^+^ Tregs was also observed in the colonic lamina propria of natural SSP mice compared with non-SSP and uninfected controls (Fig. S3 D). In this natural model, colonic Tregs isolated from SSP mice also exhibited a distinct activated phenotype, characterized by the upregulation of CD200, ICOS, GITR, and Sca-1, whereas non-SSP colonic Tregs resembled those from uninfected hosts (Fig. S3 E). Additionally, non-SSP colonic Tregs showed an intermediate phenotype in the downregulation of Rorγt and upregulation of T-bet and CXCR3 compared with both SSP and uninfected hosts. Notably, no difference in the expression of CTLA4 was observed across these three conditions (Fig. S3 E). Together, these results suggest that SSP colonic Tregs are distinguished by a significantly upregulated type 1 signature (T-bet, Cxcr3) and a sustained activation state (CD200, ICOS, GITR, Sca-1) compared with colonic Tregs from non-SSP and uninfected hosts.

We next interrogated the transcriptional signature of the T-bet^+^ Tregs to gain further insights into their functionality. The T-bet^+^ Tregs highly expressed *Tgfb1*, *Areg*, *Gzmb*, *Il2ra*, and *Ctla4*, with the modest expression of *Pdcd1*, and low expression of *Il10*, all of which were also expressed at varying levels by the *Gata3*^*hi*^ and *Rorc*^*+*^ Tregs (Fig. 3 H and Fig. S3 F). Importantly, T-bet^+^ Tregs did not express type 1 inflammatory cytokines like *Ifng* and *Tnfa* (Fig. S3 F), supporting the notion that these cells play a primary role in immune suppression during SSP infection, rather than inflammation. Altogether, these results suggest that the robust type 1 inflammatory state present in the colonic lamina propria of F1-SSP mice induces the generation of T-bet^+^ Tregs, which are highly proliferative and express genes consistent with immunosuppressive functions.

### Gzmb^+^ CD4^+^ T cells expand in the colonic lamina propria of SSP hosts in the absence of Tregs

**Figure S4.**
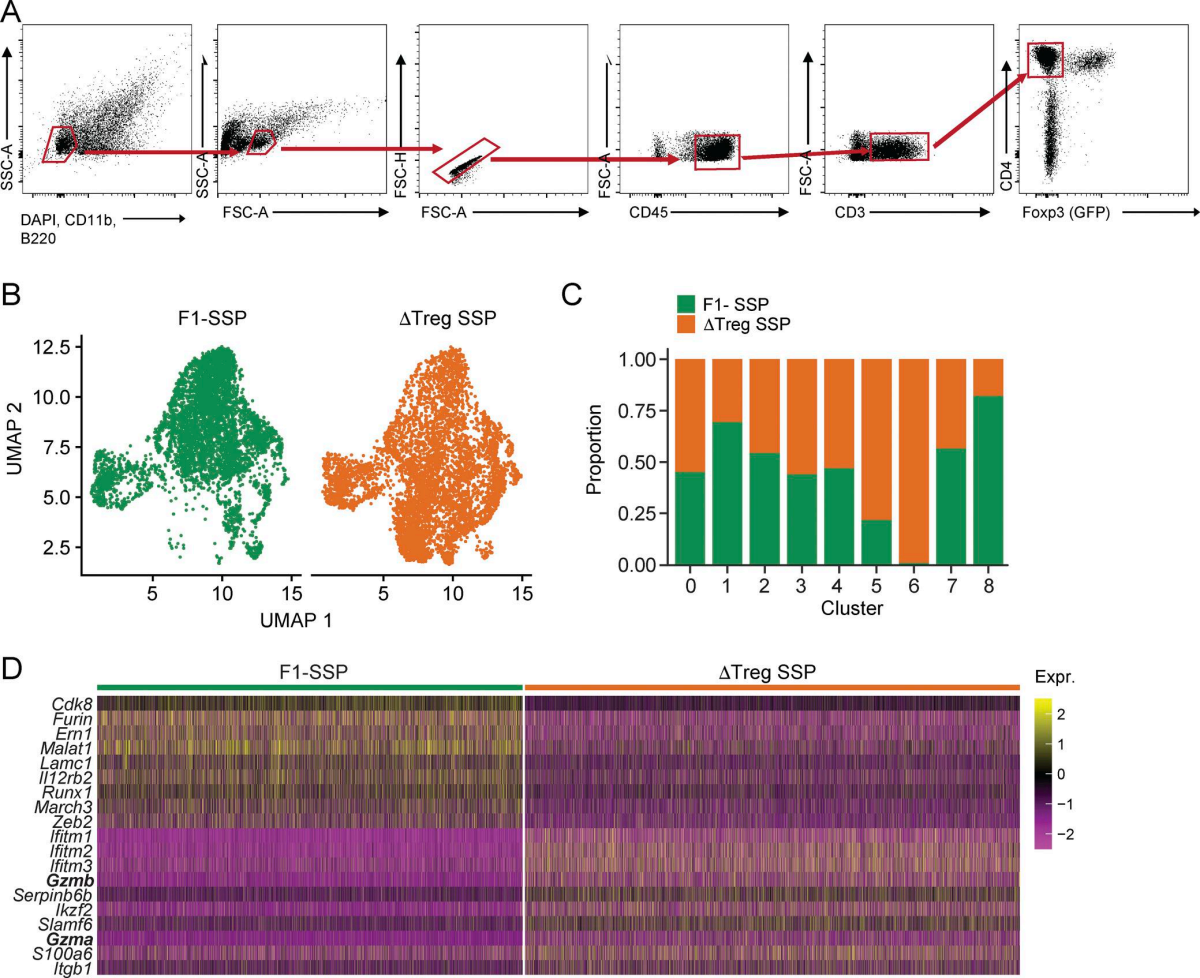
**Additional data related to the scRNA-seq analysis of colonic Foxp3** ^**−**^ **CD4** ^**+**^ **T cells isolated from F1-SSP and uninfected hosts at 28 dpi. (A)** Gating strategy used for sorting CD4^+^ T cells from colonic lamina propria of F1-SSP and ΔTreg SSP at 28 dpi. **(B)** UMAP plot showing the clustering of colonic CD4^+^ T cells based on condition (F1-SSP/ΔTreg SSP) at 28 dpi. **(C)** Barplot showing the contribution of each condition (F1-SSP/ΔTreg SSP) to the different CD4^+^ T cell clusters. **(D)** Heatmap representing the differential expression of the top 10 genes of CD4^+^ T cells generated in each condition (F1-SSP/ΔTreg SSP).

**Figure 4.**
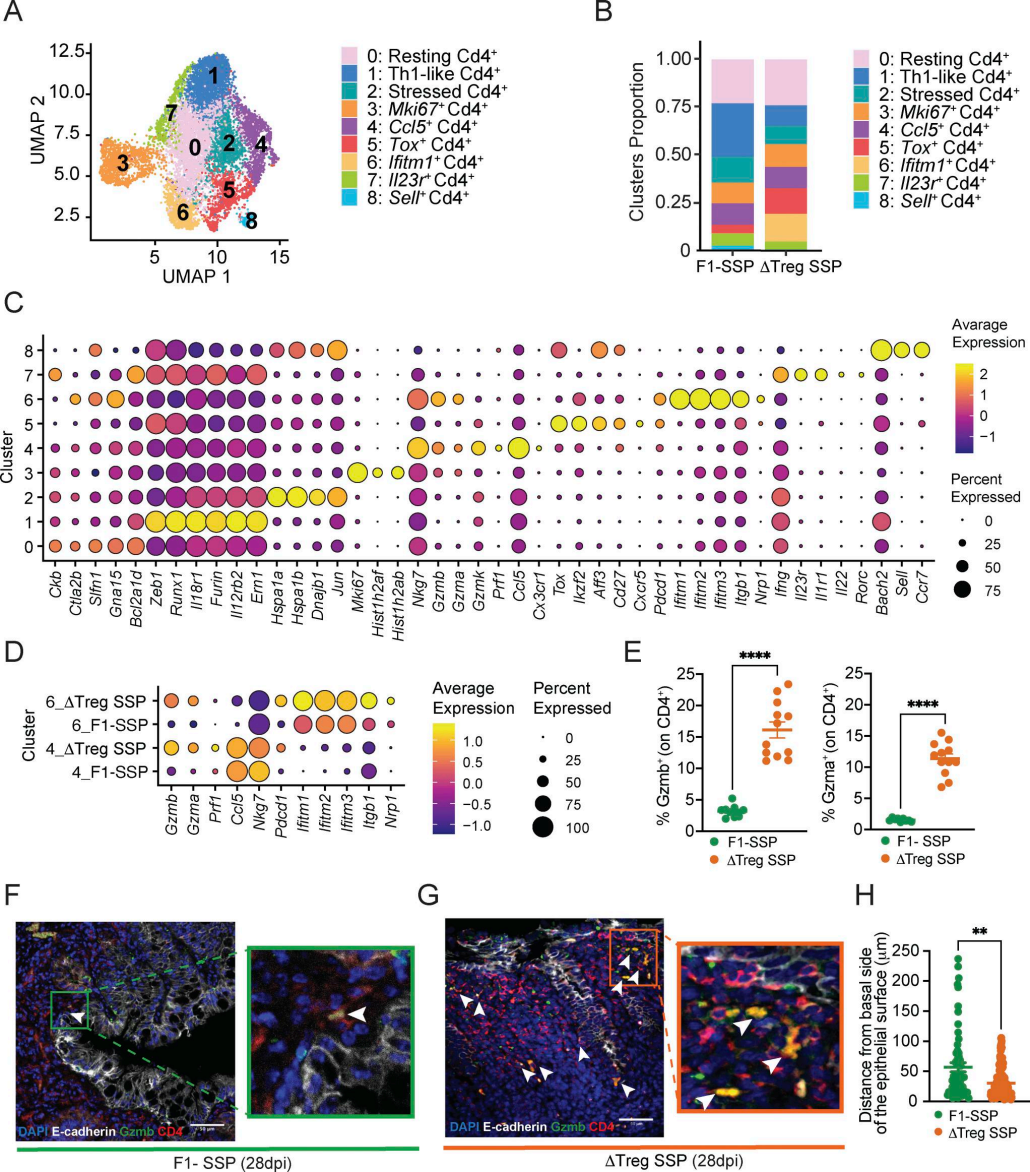
**scRNA-seq of colonic CD4** ^**+**^ **T cells reveals the upregulation of granzyme gene expression in ΔTreg SSP mice. (A)** UMAP plot of colonic CD4^+^T cell clusters (0–8) generated by merging F1-SSP and ΔTreg SSP mice at 28 dpi. **(B)** Barplot showing the proportion of each cluster of CD4^+^T cell from F1-SSP and ΔTreg SSP mice. **(C)** Dot plot representing a list of genes manually curated to identify the different gene expression signature that characterizes each cluster. The size of the dot represents the percentage of the cells in each cluster expressing a specific marker, while the color is a measure of the average expression of that gene in a specific cluster. **(D)** Dot plot representing the expression of *Gzmb*, *Gzma*, *Prf1*, *Ccl5*, *Nkg7 Pdcd1*, *Ifitm1-2-3*, *Itgb1*, and *Nrp1* in clusters 6 and 4 of F1-SSP and ΔTreg SSP mice. The size of the dot represents the percentage of the cells in each cluster expressing a specific marker, while the color is a measure of the average expression of that gene in a specific cluster. **(E)** Frequency of Gzmb^+^ and Gzma^+^ CD4^+^ T cells isolated from the colonic lamina propria of F1-SSP and ΔTreg SSP mice at 28 dpi (*n* = 10–12). Results are representative of at least two independent experiments and presented as means ± SEM. Normality was assessed by the D’Agostino–Pearson test. The Mann–Whitney U test was used to compare two groups (****P < 0.0001). **(F and G)** Representative immunofluorescence images of colonic tissues (magnification: 40×; scale bar: 50 μm) collected from F1-SSP (F) and ΔTreg SSP (G) mice, showing the close localization of Gzmb^+^ CD4^+^ T cells (white arrowheads) to the epithelial layer (E-cadherin). Sections were stained with E-cadherin (white), CD4 (red), Gzmb (green), and DAPI (blue). **(H)** Quantification of the localization of Gzmb^+^ CD4^+^ T cells as distance between them and the colonic epithelium. Results are representative of 5–10 areas per section per condition (F1-SSP and ΔTreg SSP), with at least three sections per condition. Each dot represents a Gzmb^+^ CD4^+^ T cell, and the data are presented as means ± SEM. Normality was assessed by the D’Agostino–Pearson test. The Mann–Whitney U test was used to compare two groups (**P < 0.005). See also Fig. S4.

Considering the severe intestinal inflammation and reduced survival, we suspected that immune pathology was a major cause of the increased morbidity observed upon Treg depletion in F1-SSP mice. Based on our observations that intestinal CD4^+^ T cells were robustly activated during F1-SSP infection (Figs. 1 and S1) and previous findings (Gopinath et al., 2013; Griffin and McSorley, 2011; Ingram et al., 2017; Srinivasan et al., 2004) that effector CD4^+^ T cells play crucial roles during *Salmonella* infection, we reasoned that the absence of Tregs resulted in the hyperactivation of CD4^+^ T cells, which underlie the pathology. To address this hypothesis, we employed an unbiased approach performing scRNA-seq of CD4^+^ T cells sorted from the colonic lamina propria of F1-SSP hosts, with or without Tregs (Fig. S4 A). After quality controls, we obtained 17,172 cells, which were then categorized into nine clusters (Fig. 4 A) with distinct distributions between F1-SSP and ΔTreg SSP hosts (Fig. 4 B; and Fig. S4, B and C). These clusters were then named based on their gene expression profiles (Fig. 4 C).

Comparison of all the clusters identified revealed that some were similarly present in both conditions (clusters 0, 2, 3, 4, and 7; Fig. S4, B and C), that clusters 1 and 8 were preferentially present in F1-SSP mice, and that clusters 5 and 6 were almost exclusively present in ΔTreg SSP mice (Fig. S4, B and C). Cluster 5 was defined by the expression of genes like *Tox*, *Cxcr5*, and *Pdcd1*, consistent with T follicular helper-like cells, which are typically localized in colonic patches or isolated lymphoid follicles and regulate IgA production during *Salmonella* infection (Martinoli et al., 2007; Vazquez-Torres et al., 1999). Cluster 6 was characterized by the expression of the IFN-γ–induced genes *Ifitm1*, *Ifitm2*, and *Ifitm3*, which have been reported to be associated with inflammatory bowel diseases (Wu et al., 2007), alongside with the integrin *Itgb1* (Pawlak et al., 2022).

Interestingly, differential gene expression analysis between the two conditions (F1-SSP versus ΔTreg SSP), irrespective of clustering, highlighted granzyme A and B as highly expressed genes in the absence of Tregs during *Salmonella* infection compared with F1-SSP hosts (Fig. S4 D). *Gzma*, *Gzmb*, *and Prf1* transcripts were upregulated in clusters 6 and 4 from ΔTreg SSP mice, indicating the development of a cytotoxic profile in colonic CD4^+^ T cells upon Treg depletion (Fig. 4 D). We confirmed that colonic CD4^+^ T cells in ΔTreg SSP mice displayed the elevated expression of granzyme A and B proteins, which was minimally detectable in CD4^+^ T cells from F1-SSP hosts (Fig. 4 E). Immunofluorescence imaging further revealed a significant increase in Gzmb^+^ CD4^+^ T cells in ΔTreg SSP mice, which were localized more closely to the epithelial barrier compared with F1-SSP mice (Fig. 4, F–H). Collectively, our findings demonstrate that Treg depletion during chronic *Salmonella* infection triggers an expansion of colonic cytotoxic CD4^+^ T cells that localize near the colonic epithelium, where they likely weaken barrier integrity, and exacerbate immunopathology and gut permeability.

### CD4^+^ T cell depletion restores tolerance in Treg-depleted superspreader hosts

**Figure 5.**
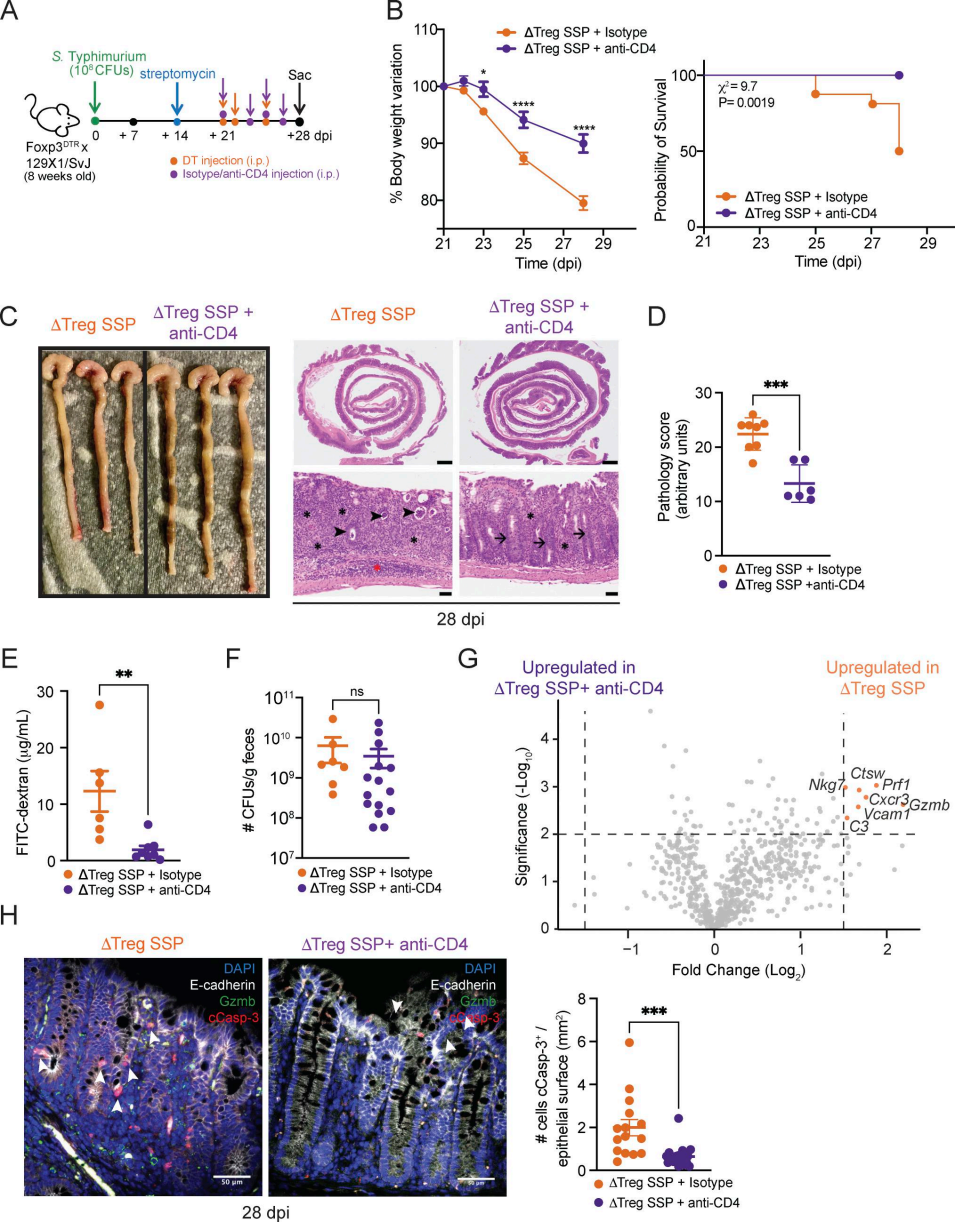
**Depletion of CD4** ^**+**^ **T cells, but not CD8** ^**+**^ **T cells, ameliorates intestinal immune pathology of SSP hosts in the absence of Tregs. (A)** Schematic representation of the experimental design. F1-Foxp3^DTR^ mice (*n* = 10) were infected with *Salmonella* Typhimurium SL1344 (10^8^ CFUs; *S*Tm), treated with streptomycin to induce SSP at 14 dpi, and injected (i.p.) with DT and anti-CD4 or isotype antibody to deplete CD4^+^ T cells, as indicated. **(B)** Percentage of body change variation (left) and probability of survival (right) of ΔTreg SSP (orange circle) and CD4-depleted ΔTreg SSP (purple circle) mice starting at 21 dpi (*n* = 10). **(C)** Representative images (left) and H&E staining of colon collected from ΔTreg SSP and CD4-depleted ΔTreg SSP at 28 dpi, as indicated. Magnification: upper panels = 1.25×; lower panels = 20×. Scale bars: upper panels = 1.0 mm; lower panels = 50 μm. Inflammation in the lamina propria (black asterisks) and the submucosa (red asterisk). Luminal cell debris (black arrowheads), goblet cells (black arrows). **(D)** Pathology scores assigned to colonic tissue sections of ΔTreg SSP (orange circle) and CD4-depleted ΔTreg SSP (purple circle, *n* = 4–8). **(E)** FITC-dextran detected in the serum of ΔTreg SSP and CD4-depleted ΔTreg SSP at 28 dpi (*n* = 6–8). **(F)***Salmonella* CFUs measured in the feces of ΔTreg SSP and CD4-depleted ΔTreg SSP at 28 dpi (*n* = 8–15). **(G)** Volcano plot of differentially expressed genes between ΔTreg SSP and CD4-depleted ΔTreg SSP colonic tissues at 28 dpi. Orange dots indicate the genes that were differentially upregulated in the colon collected from ΔTreg SSP mice compared with CD4-depleted ΔTreg SSP. **(H)** Representative immunofluorescence images of colonic tissues (magnification 40×; scale bar: 50 μm) collected from ΔTreg SSP (left) and CD4-depleted ΔTreg SSP (right) mice, showing the localization of cCasp-3^+^ cells (white arrowheads) mostly in epithelium layer (E-cadherin^+^). Sections were stained with E-cadherin (white), cCasp-3 (red), Gzmb (green), and DAPI (blue). Numbers of cCasp-3^+^ E-cadherin^+^ cells localized in the epithelium in at least five areas per section per condition (ΔTreg SSP and ΔTreg SSP + anti-CD4), with at least three sections per condition. Each dot represents a cCasp-3^+^ E-cadherin^+^ cell, and the data are presented as means ± SEM. Normality was assessed by the D’Agostino–Pearson test. The Mann–Whitney U test was used to compare two groups (***P <0.005). Results in panels B and D–F are representative of at least two independent experiments and presented as means ± SEM. Normality was assessed by the D’Agostino–Pearson test. The Mann–Whitney U test (D–F and H) was used to compare two groups. Two-way ANOVA (B) followed by post hoc Sidak’s test was performed for multiple groups comparisons (****P < 0.0001; ***P < 0.001; **P < 0.01; *P < 0.05; ns = not significant). A log-rank (Mantel–Cox) (B) test was used to compare survival probability between the two groups. See also Fig. S5; and Tables S1 and S2.

**Figure S5.**
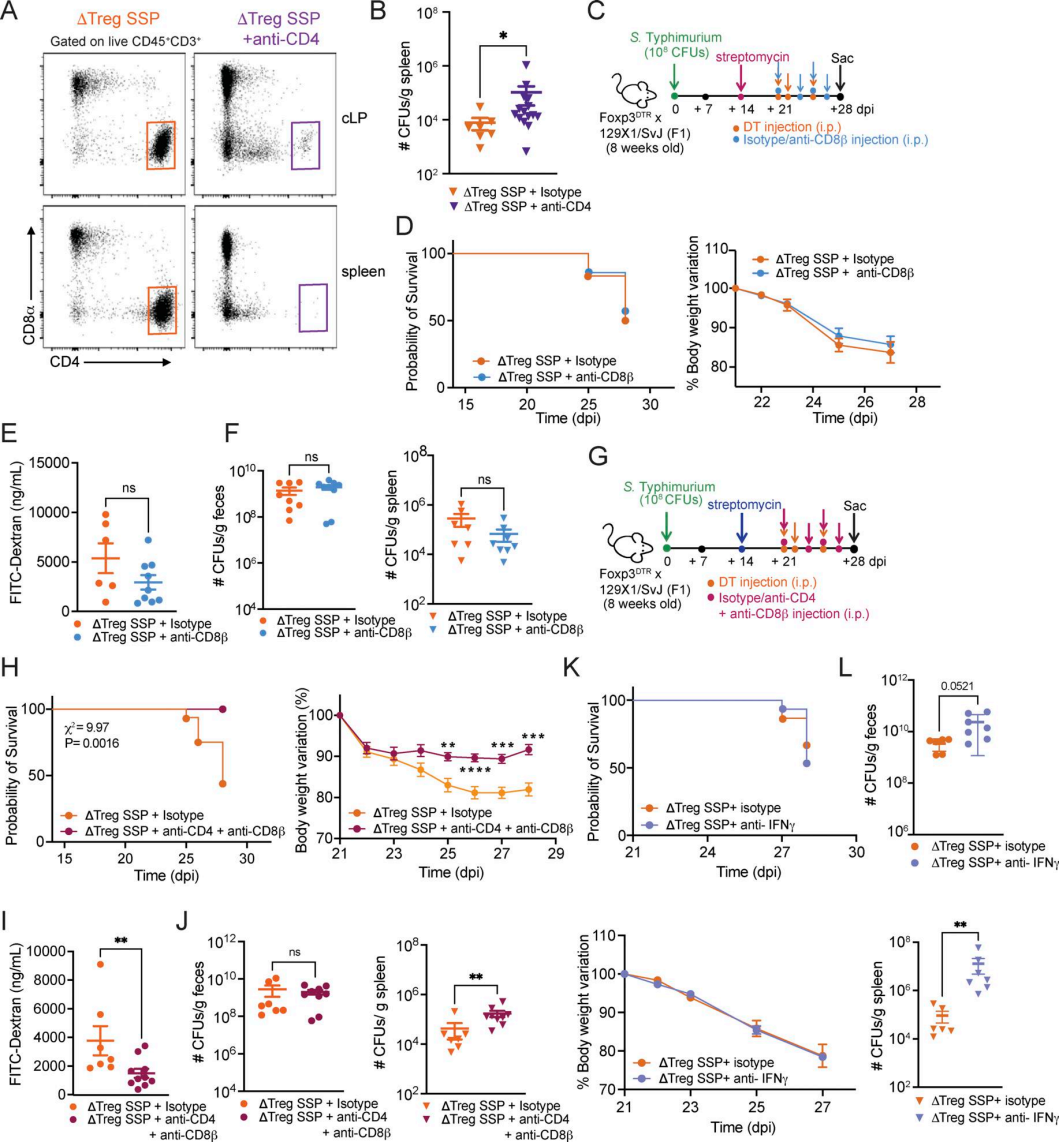
**Depletion of either CD8** ^**+**^ **T cells or the pro-inflammatory cytokine IFN-γ does not affect morbidity and mortality in ΔTreg SSP mice. (A)** Representative FACS plots showing CD4^+^ T cell depletion in the colonic lamina propria (cLP; top) and spleen (bottom) of ΔTreg SSP mice. **(B)***Salmonella* CFUs measured in the spleen of ΔTreg SSP and CD4-depleted ΔTreg SSP at 28 dpi (*n* = 8–15). **(C)** Schematic representation of the experimental design. F1-Foxp3^DTR^ mice (*n* = 10) were infected with *Salmonella* Typhimurium SL1344 (10^8^ CFUs; *S*Tm), treated with streptomycin to induce SSP at 14 dpi, and injected (i.p.) with DT and anti-CD8β/isotype antibodies to deplete CD8^+^ T cells, as indicated. **(D)** Survival curve (left) and percentage of body weight variation (right) of ΔTreg SSP mice treated with anti-CD8β (turquoise circle) or isotype antibodies (orange circle) starting at day 21 after infection (*n* = 10). **(E)** FITC-dextran (ng/ml) measured in the serum of ΔTreg SSP and CD8-depleted ΔTreg SSP at 28 dpi (*n* = 6–10). **(F)***Salmonella* CFUs measured in the fecal content (left) and spleen (right) of ΔTreg SSP and CD8-depleted ΔTreg SSP at 28 dpi (*n* = 10). **(G)** Schematic representation of the experimental design. F1-Foxp3^DTR^ mice (*n* = 10) were infected with *Salmonella* Typhimurium SL1344 (10^8^ CFUs; *S*Tm), treated with streptomycin to induce SSP at 14 dpi, and injected (i.p.) with DT and anti-CD4 and CD8β or isotype antibodies to deplete CD4^+^ and CD8^+^ T cells, as indicated. **(H)** Survival curve (left) and percentage of body weight variation (right) of ΔTreg SSP mice treated with anti-CD4 and CD8β (magenta circle) or isotype antibodies (orange circle) starting at day 21 after infection (*n*= 10–15). A log-rank (Mantel–Cox) test was used to compare survival probability between the two groups. **(I)** FITC-dextran (ng/ml) measured in the serum of ΔTreg SSP and CD4-CD8-depleted ΔTreg SSP at 28 dpi (*n* = 6–10). **(J)***S*Tm CFUs measured in the fecal content (left) and spleen (right) of ΔTreg SSP (orange) and CD4- and CD8-depleted ΔTreg SSP (magenta) at 28 dpi (*n* = 10). **(K)** Survival curve (top) and percentage of body weight variation (bottom) of ΔTreg SSP mice treated with anti-IFN-γ (lilac circle) or isotype antibodies (orange circle) starting at day 21 after infection (*n* = 10). **(L)***S*Tm CFUs measured in the fecal content (top) and spleen (bottom) of ΔTreg SSP mice treated with anti-IFN-γ (lilac) or isotype antibodies (orange) at 28 dpi (*n* = 10). Results are representative of at least two independent experiments and presented as means ± SEM. Normality was assessed by the D’Agostino–Pearson test. The Mann–Whitney U (B, E, F, I, J, and L) test was used to compare two groups (****P < 0.0001; ***P < 0.001; **P < 0.01; *P < 0.05, ns = not significant). Two-way ANOVA (D, H, and K) followed by post hoc Sidak’s test was performed for multiple groups comparisons. A log-rank (Mantel–Cox) (H) test was used to compare survival probability between the two groups.

We hypothesized that effector CD4^+^ T cells, which exhibit a cytotoxic signature, were responsible for the exacerbated immunopathology observed in Treg-depleted mice. To test this, we depleted CD4^+^ T cells by administering anti-CD4 antibody (or isotype control antibody) to ΔTreg SSP mice (Fig. 5 A) and a robust depletion efficacy was confirmed by flow cytometry (Fig. S5 A). Strikingly, CD4-depleted ΔTreg SSP mice had reduced weight loss and increased survival (Fig. 5 B), indicating a rescue of the ΔTreg SSP phenotype. CD4-depleted ΔTreg SSP mice also exhibited a reduced colonic pathological score (Fig. 5, C and D) and decreased damage to the intestinal epithelial barrier as measured by FITC-dextran in the serum, compared with controls (Fig. 5 E). Surprisingly, CD4^+^ T cell depletion did not impact *Salmonella* burden in the gut (Fig. 5 F), although an increase in *Salmonella* CFUs was observed in the spleen (Fig. S5 B). This is likely due to the depletion of splenic CD4^+^ T cells, which act as the major source of IFN-γ at this site, and clearly highlights the distinctive qualities of pathogen–immune interactions at mucosal barriers compared with lymphoid organs such as the spleen.

To validate the predominant role of CD4^+^T cells as primary contributors to the intestinal immunopathology, we administered anti-CD8β (or isotype control) antibodies to deplete CD8^+^ T cells in ΔTreg SSP mice (Fig. S5 C). Depletion of CD8^+^ T cells did not affect weight loss, survival, or gut permeability in ΔTreg SSP compared with the isotype controls (Fig. S5, D and E). No changes in the fecal shedding levels or in splenic CFUs between the two groups were detected (Fig. S5 F). Additionally, when we simultaneously depleted CD4^+^ and CD8^+^ T cells (Fig. S5 G), ΔTreg SSP hosts devoid of both subsets exhibited a phenotype resembling the improved outcomes observed with CD4^+^T cell depletion alone (Fig. S5, H and I). As expected, no differences were observed in the fecal shedding levels between CD4^−^CD8^−^-depleted ΔTreg SSP hosts and the isotype controls, while *Salmonella* CFUs in the spleen increased (Fig. S5 J). These data indicate that CD8^+^ T cells do not contribute significantly to either the severity of intestinal pathology or the control of *S*Tm burden in ΔTreg SSP mice.

Finally, given the heightened production of IFN-γ and granzymes (Fig. 1 G; and Fig. 4, D and E) by CD4^+^ T cells during *Salmonella* infection, we examined their role in exacerbating the immunopathology. IFN-γ neutralization (Fig. S5 K) did not ameliorate the increased morbidity and mortality observed in ΔTreg SSP mice but did exacerbate pathogen burden in both the feces and spleen (Fig. S5 L), indicating that IFN-γ was not a primary driver of the observed immunopathology. NanoString gene expression analysis of the whole colonic tissue revealed that the upregulation of genes such as *Gzmb*, *Prf1*, *Cxcr3*, and *Nkg7* in ΔTreg SSP was strongly reduced upon depletion of CD4^+^ T cells (Fig. 5 G), supporting a crucial role of cytotoxic granzymes in immunopathology. Immunofluorescence imaging of the colon revealed a striking increase in Gzmb^+^ CD4^+^ T cells near the epithelial barrier in ΔTreg SSP mice (Fig. 4, F–H), accompanied by elevated epithelial cell apoptosis (cleaved caspase-3^+^ E-cadherin^+^/CD324^+^; Fig. 5 H) compared with F1-SSP controls. Supporting the hypothesis that cytotoxic CD4^+^ T cells are involved in damaging the tissue, CD4^+^ T cell depletion in ΔTreg SSP mice significantly reduced epithelial cell apoptosis compared with controls (Fig. 5 H). Together, these findings highlight that Tregs restrain the expansion of cytotoxic CD4^+^ T cells in SSP hosts. In their absence, as seen in ΔTreg SSP mice, unchecked cytotoxic CD4^+^ T cells are associated with a compromised mucosal barrier, increased immunopathology, and exacerbated host morbidity—without altering pathogen burden.

## Discussion

Understanding the mechanisms that sustain long-term tolerance in superspreader hosts is crucial, as these hosts are the primary agents for most of disease transmission. Here, we show that Tregs are essential for maintaining the asymptomatic state of superspreader hosts during chronic *Salmonella* infection. The SSP state induced transcriptionally distinct populations of Tregs in the colon, characterized by the expression of T-bet and type 1 inflammatory genes. In the absence of Tregs, CD4^+^ T cells caused severe immunopathology, which could be reversed by depletion of these cells. Mechanistically, the upregulation of a cytotoxic program in CD4^+^ T cells correlated with increased apoptosis of colonic epithelial cells, loss of intestinal barrier integrity, and colonic inflammation. However, the lack of specific genetic tools prevented a definitive proof of a direct role of this cytotoxic program in the colonic tissue damage. Overall, this study elucidates host pathways, which are essential for tolerance to chronic infection and which intracellular pathogens exploit to thrive within their hosts and to use them as carriers for dissemination.

Tregs are a specialized lymphocyte population dedicated to suppressing uncontrolled and excessive inflammatory responses of the host to self- and environmental antigens, commensal microbiota, infectious agents, and tumors (Rudensky, 2011; Sakaguchi et al., 2008). Previous studies using various infection models have shown that Treg depletion generally leads to heightened T cell activation and improved control of pathogen burden (Abel et al., 2016; Arnold et al., 2011; Galdino et al., 2018; Johanns et al., 2010; Scott-Browne et al., 2007). In the context of chronic *Salmonella* infection, via the intravenous route, Tregs have been demonstrated to play a time-dependent role in modulating pathogen clearance in lymphoid organs (Johanns et al., 2010). In our model of *Salmonella* infection through the physiological oral route, we demonstrate that Treg depletion significantly disrupts colonic tolerance without affecting pathogen burden. These findings highlight the importance of the tissue environment where the immune system encounters the pathogen (e.g., sterile sites vs. mucosal barriers) as a key factor in shaping host responses and determining distinct health outcomes.

Additionally, several studies have reported increased pathogen burden in the absence of Tregs due to impaired T cell differentiation (Soerens et al., 2016; Wang et al., 2014). However, despite the dramatic morbidity and reduced survival observed upon Treg ablation during infection, we detected no differences in bacterial burden in feces or spleen between ΔTreg SSP and Treg-sufficient SSP mice. Interestingly, while CD4^+^ T cell depletion in ΔTreg SSP mice led to increased bacterial burden in the spleen, fecal *Salmonella* CFUs remained unchanged. This finding underlines that host tolerance mechanisms at the mucosal sites differ fundamentally from systemic immune responses during enteric infections. Therefore, investigating host responses to chronic *Salmonella* infection at physiologically relevant mucosal barriers can uncover novel insights that may not be revealed by studying systemic organs alone. In line with this, our group and others (Diaz-Ochoa et al., 2016; Ruddle et al., 2023; Shelton et al., 2022; Winter et al., 2010; Yoo et al., 2024) have shown that *Salmonella* employs distinct metabolic strategies in the gut, enabling it to flourish extracellularly within an inflamed microenvironment teeming with commensal microbes. A critical unanswered question remains: do *Salmonella* or the adapted commensal microbiota during chronic infection directly modulate the intestinal Treg response? This interaction may play a pivotal role in maintaining the host’s asymptomatic state while facilitating pathogen persistence and environmental spread. Addressing this question is essential to unraveling the complex dynamics of host–pathogen–microbiota interplay during chronic infections.

scRNA-seq analysis revealed a distinct transcriptional profile in colonic Tregs from SSP hosts compared with those under homeostatic conditions. Strikingly, the transcription factor T-bet was upregulated almost exclusively in SSP hosts. These T-bet^+^ Tregs displayed heightened proliferation, suggesting they actively respond to environmental signals and expand to sustain a tolerogenic state. Prior studies (Koch et al., 2009; Levine et al., 2017) have demonstrated that T-bet expression in Tregs is induced by an IFN-γ–enriched environment, enabling their localization to sites of Th1-driven inflammation, where they exert suppressive functions. Our findings align with a model where the IFN-γ–rich environment in the SSP colon promotes the development of T-bet^+^ Tregs, which restrain a pathogenic CD4^+^ T cell response and thereby maintain the asymptomatic state. The specificity of these T-bet^+^ Tregs remains an open question. We were unable to detect any Tregs specific for the immunogenic peptide 2W1S_56-68_ expressed by *Salmonella*. One possibility is that the 2W1S peptide is not ideal for Treg formation and that other *Salmonella* antigens dominate the expansion of *Salmonella*-specific Tregs. Another intriguing possibility is that these expanded Tregs are specific for commensal microbes, food, or self-antigens encountered in the gut. Further work is also needed to determine whether these T-bet^+^ Tregs are derived from preexisting gut CD4^+^ T cell populations or whether they are generated peripherally *de novo* in response to infection. Addressing these questions is essential for understanding the antigenic drivers and the origins of T-bet^+^ Tregs during chronic infection.

Our investigation into the CD4^+^ effector T cell populations driving immunopathology in Treg-depleted SSP hosts revealed a significant upregulation of cytotoxic genes. We found that CD4^+^ T cells producing granzyme B predominantly localized near the intestinal barrier, which had more apoptotic epithelial cells, indicating that cytotoxic CD4^+^ T cells are likely involved in damaging the colonic epithelium. Remarkably, depletion of CD4^+^ T cells alleviated the colonic immunopathology observed in ΔTreg SSP. Although CD8^+^ T cells also express granzyme genes, their depletion did not rescue the immunopathology observed in ΔTreg SSP. This is contrary to observations in viral infections, where CD8^+^ T cell depletion typically reduces immunopathology (Labarta-Bajo et al., 2020), and suggests a unique role of colonic CD4^+^ T cells in mucosal *Salmonella* infection. While multiple lines of evidence in this study (Figs. 4 and 5) implicate cytotoxic CD4^+^T cells in the heightened gut pathology, a limitation of this study is the absence of specific genetic tools in the 129X1/SvJ mouse background. Therefore, additional experiments and development of novel mouse models are needed to conclusively establish the role of cytotoxic CD4^+^T cells in the enhanced gut pathology.

The precise mechanism by which colonic Tregs restrain cytotoxic CD4^+^ T cell responses during *Salmonella* infection remains unclear. Recent studies suggest that in the absence of Tregs, elevated IL-2 levels in the tumor microenvironment promote cytotoxic activity in T helper cells through the transcription factor Blimp-1 (Śledzińska et al., 2020). In the highly pro-inflammatory colonic tissue of SSP mice, elevated IL-2 levels may similarly drive the expansion of cytotoxic CD4^+^ T cells, resulting in increased epithelial damage when Tregs are depleted. However, Tregs in SSP mice express other genes, which could promote immune suppression, including *Tgfb1*, *Areg*, *Ctla4*, and to a lesser extent *Il10* and *Pdcd1*. They also express *Gzmb*, which may suppress cytotoxic CD4^+^ T cells through direct killing mechanisms of effector CD4^+^ T cells. A critical question remains: what antigens are driving the cytotoxic CD4^+^ T cells—self, commensal, or pathogen-derived? Determining their TCR specificity will provide insight into why these cells target the colonic epithelium. Future mechanistic studies using the SSP model will be instrumental in defining how Tregs enforce a disease-tolerant state and what the specific role of cytotoxic CD4^+^ T cells is in the enhancement of the gut immunopathology. Such insights could reveal therapeutic pathways broadly applicable to inflammatory bowel disorders and inform strategies for immunotherapy.

## Materials and methods

### Mouse strains and husbandry

129X1/SvJ (strain #: 000691) and FOXP3^DTR^ (B6.129(Cg)-Foxp3^tm3(DTR/GFP) Ayr^/J; strain #: 016958) were purchased from The Jackson Laboratory. Great (B6.129S4-Ifng^tm3.1Lky^/J) mice were generously provided by Dr. Richard Locksley’s laboratory at University of California, San Francisco (UCSF), San Francisco, CA, USA. F1 mice were generated by breeding all the mentioned reporter mouse strains on a C57BL/6 background with 129X1/SvJ. In the generation of F1-Foxp3^DTR^ mice, the *Foxp3* gene, being X-linked, results in male progeny (129X1/SvJ × C57BL/6) carrying only one copy of the *Foxp3*^*DTR*^ gene (F1-Foxp3^DTR^). Upon treatment with DT, male progeny undergo complete Treg depletion (ΔTreg), whereas the female progeny, which retain one functional copy of the *Foxp3* gene, experience only partial Treg depletion. Therefore, for our experiments, we exclusively utilized male progeny either treated with DT (ΔTreg) or untreated (F1), as indicated.

Mice were housed under specific pathogen–free conditions in filter-top cages that were changed biweekly by research personnel. Water and food were provided ad libitum. Mice were acclimated for at least 1 wk at the Stanford Animal Biohazard Research Facility prior to experimentation. All animal procedures were approved by Stanford University Administrative Panel on Laboratory Animal Care and overseen by the Institutional Animal Care and Use Committee under Protocol ID 12826.

### Bacterial strains and growth conditions

*S*Tm SL1344 and *S*Tm SL1344-2W1S (generously provided by Dr. Stephen McSorley’s laboratory at University of California, Davis, Davis, CA, USA) were maintained aerobically on LB agar supplemented with 200 μg/ml streptomycin and grown aerobically for 16 h at 37°C on a shaker (200 × *g*).

### Mouse infections

8–12-wk-old mice age- and gender-matched were starved overnight and infected with *Salmonella* strains by feeding the mice with 20 ml of the bacterial suspension (5 × 10^9^ CFU/ml) directly into the mouth using a pipette. Shedding levels were monitored weekly by collecting fecal pellets in tubes containing 500 ml of PBS. The samples were weighed, serially diluted, and spot-plated in duplicates on LB agar plates for CFU counting, and the counts were adjusted by the weight of the pellets. Mice were humanely euthanized at the indicated time points. Spleen, liver, and mesenteric lymph nodes were collected and dissociated in PBS, and serially diluted for CFU count. The counts were adjusted by the organ weight.

To induce the SSP state, mice received 5 mg of streptomycin 14 days after *S*Tm infection, by drinking from a pipette tip.

### In vivo treatments

F1 mice were intraperitoneally (i.p.) injected with 50 μg/kg DT (cat. no. D0564; Sigma-Aldrich) on days 21 and 22 after infection and with 10 μg/kg DT on day 25 after infection.

F1 mice were i.p. injected with anti-CD4 and/or anti-CD8β, or anti-IFN-γ monoclonal antibodies. 100 μg of either/both InVivoMAb anti-mouse CD8β (clone Lyt3.2; BE0223; BioXcell) and InVivoMAb anti-mouse CD4 (clone GK1.5; BE0003-1; BioXcell), or InVivoMAb anti-mouse IFN-γ (clone XMG1.2; BE0055; BioXcell) or isotype-matched control antibody (rat IgG2a isotype control, BP0089; rat IgG2b isotype control, BE0090; rat IgG1 isotype control, BE0088; BioXcell, respectively) was diluted in PBS and administered on 21, 23, 25, and 27 dpi.

### Intestinal permeability assay

Mice were administered FITC-dextran (4,000 Da; Sigma-Aldrich) by oral gavage at 0.44 mg per gram of body weight. 4 h later, mice were humanely euthanized, and blood was collected. FITC-dextran concentrations in the serum were measured via fluorescence spectrophotometry on Synergy HTX with an excitation wavelength of 485 nm (20-nm bandwidth) and an emission wavelength of 528 nm (20-nm bandwidth). A standard curve was prepared with a serial dilution of 100 μg/ml of FITC-dextran in PBS.

### Colonic lamina propria isolation

The colon was harvested, cut longitudinally, and washed with Hanks’ balanced salt solution (HBSS, Gibco) for removing luminal content. Intestinal tissues were incubated twice with HBSS/HEPES supplemented with 5 mM DTT, 5 mM EDTA, and 5% vol/vol bovine calf serum (BCS) at 37°C for 15 min, and washed with HBSS/HEPES supplemented with 2% vol/vol BCS at 37°C for 5 min. Then, the colonic tissues were incubated in RPMI containing 0.167 mg/ml Liberase TL (Roche), 0.25 mg/ml DNase I (Sigma-Aldrich), and 5% BCS on a shaker at 37°C for 30 min. Finally, they were dissociated with gentleMACS Dissociator (Mylteni). Immune cells were enriched using a 40–70% PERCOLL gradient (GE Healthcare) and resuspended in FACS buffer (PBS 1X, 2 mM EDTA, and 2% vol/vol BCS).

### Flow cytometry

Single-cell suspensions were incubated for 5 min on ice with Fc Block (TruStain FcX anti-mouse CD16/32; BioLegend) and then stained on ice for 20 min with a cocktail of antibodies for surface antigens in Fc Block. The following antibodies were used: CD45-APC/Cy7 or Pacific Blue (clone 30-F11); CD11b-BV785 (clone M1/70); I-A/I-E-PE (clone M5/114.15.2); Ly6C-BV711 or PerCP/Cy5.5 (clone HK1.4); Ly6G-BUV395 (clone 1A8); CD64-BV605 (clone X54-5/7.1); CX3CR1-BV650 (clone SA011F11); CD206/MMR–Alexa Fluor 488 (clone C068C2); F4/80-APC (clone BM8); CD11c-PE/Cy7 (N418); CD3–Alexa Fluor 700 (clone 17A2); CD8α-BV711 (clone 53-6.7); CD4-PECF594 or CD4-BUV395 (clone RM4-5); TCRγδ-BV605 (clone GL3); CD44-BV785 (clone IM7); CD62L-PE/Cy7 (clone MEL-14); CD200-PE-Dazzle (clone OX-90); Ly6A/E-PerCP/Cy5.5 (clone D7); CD278-BV711 (clone C398.4A); CD152-BV605 (clone UC10-4B9); CXCR3-BV605 (clone CXCR3-173); GITR-PE/Cy7 (clone DTA-1); Ki-67-BUV395 (clone B56); GATA3-PerCP/Cy5.5 or BUV395 (clone L50-823); RORγt-PE (clone Q31-378) or APC (clone AFKJS-9); T-bet-BV421 (clone O4-46); FoxP3-AF647 (clone 150D); granzyme A–PE (clone 3G8.5); and granzyme B–PE/Cy7 (clone QA16A02). For tetramer staining, cells were incubated with PE-conjugated 2W1S::I-A^b^ MHCII tetramer (provided by the National Institutes of Health Tetramer Core Facility) for 1 h in Fc Block before staining for surface antigens, when required. Dead cells were excluded using the LIVE/DEAD Fixable Aqua Dead cell stain kit (Thermo Fisher Scientific).

For transcription factor staining, cells were fixed and permeabilized using the Foxp3 staining buffer set (eBioscience) according to the manufacturer’s instructions. For cytokine staining, cells were fixed and permeabilized using the Cytofix/Cytoperm buffer set (BD Biosciences) according to the manufacturer’s protocol. Flow cytometry acquisition was performed on Symphony (BD Biosciences) with BD FACSDiva software. All FACS data were analyzed using FlowJo v.10.10.0 software (Tree Star).

### Histopathological analysis and microscopy

Colons were harvested, washed with cold PBS, opened longitudinally, and fixed in phosphate-buffered formalin for 48 h at 4°C. After fixation, the linearized colon was rolled into a “Swiss roll.” Tissues were routinely processed, embedded in paraffin, sectioned at 5.0 mm, and routinely stained with hematoxylin and eosin (H&E) or processed for immunofluorescence.

Tissues stained with H&E were visualized with an Olympus BX43 upright bright-field microscope, and images were captured using an Olympus DP27 camera and cellSens software. Tissues were assessed blindly by a board-certified veterinary pathologist. Four criteria were used to grade each section of intestine, with several modifications from the scheme described by Li et al. (2014): (1) percent area affected by ulceration (defined as the absence of epithelial lining exposing the lamina propria or deeper layers), (2) severity of inflammation (defined as the presence of inflammatory cells at various layers of the intestinal wall; includes changes in the paucity of the crypt profiles), (3) percent area involved/affected by inflammation, and (4) percent area affected by edema and/or fibrin exudation (defined as the expansion of the connective tissue with edema fluid and/or fibrin, with or without inflammatory cells). For each tissue, each criterion was graded thrice, and the scored was averaged. When considering inflammation, and to more accurately reflect how much tissue was affected with a specific degree of severity, a multiplier score was obtained by multiplying the severity of inflammation by the percent area affected. For each tissue, the final score was calculated as follows:

X=[% ulceration]+[(severity of inflammation)*(% inflammation)]+[% edema & fibrin exudation].

With this, the minimum score for each tissue was 0, and the maximum possible score was 28. The grading scheme and definitions of each score are shown in Table S2. For the different criteria, the histologic assessments and categorizations are based on those areas that show the most profound changes.

For immunofluorescence, paraffin sections were deparaffinized in xylene and rehydrated in ethanol. Antigen retrieval was performed in a Tris-EDTA + 0.05 % Tween-20 (pH 9) buffer under boiling water and increased pressure for 10 min. The slides were then incubated with blocking buffer (PBS+ 0.25% Triton X-100 + 5% BCS) for 1 h at room temperature. The slides were stained with rabbit anti-mouse CD4 (clone RM1013), goat anti-mouse granzyme B, anti-mouse CD324/E-cadherin–AF647 (clone DECMA-1), and rabbit anti-mouse cleaved caspase-3 overnight at 4°C, as indicated. Slides were washed twice with wash buffer (PBS + 0.25% Triton X-100), then incubated for 1 h at room temperature with the respective secondary antibodies and DAPI. The slides were mounted with Prolong Diamond (Life Technologies). Images were collected using a 40× objective on a Zeiss LSM 700 confocal microscope (Carl Zeiss) with ZEN 2.3 SP1 software (Carl Zeiss) and processed using Volocity Image Analysis software (Quorum Technologies). Cell counts and distance measures were performed using ImageJ software.

### Sample preparation for scRNA-seq

Single-cell suspensions of the colonic lamina propria collected from three mice (age- and gender-matched littermates) were pooled together per each condition and then sorted on a BD FACSAria cell sorter, as indicated (Figs. 3 and 4). The viability of sorted cells was checked using trypan blue staining. Samples had viability >80%. Cells were resuspended to a concentration of 1,000 cells/μl. Manufacturer’s instructions were followed without any significant modifications to capture the cells, generate next-generation sequencing (NGS) libraries, and perform sequencing. Briefly, cells were captured on a 10x Chromium single-cell instrument (10x Genomics) and subjected to single-cell barcoded cDNA synthesis using NextGem V3.1 chemistry (10x Genomics). Purified cDNA was amplified and subjected to enzymatic fragmentation, end-repair, A-tailing, adapter ligation, and sample indexing to generate sequencing-ready libraries. Libraries were assessed by electrophoresis on a BioAnalyzer 2100 instrument (Agilent) and quantitative PCR on a CFX96 (Bio-Rad) instrument prior to sequencing. Libraries were sequenced on a NovaSeq 6000 sequencer (Illumina) for optimal recommended read depth. Raw sequencing data were parsed through Cell Ranger (10x Genomics) to generate FASTQ files and facilitate further analysis.

### scRNA-seq analysis

scRNA-seq analysis was conducted using the Seurat package (v4.1.1) in R. Quality control was performed to remove cells with mitochondrial content exceeding 5%, as well as those containing <1,000 or >7,000 genes per cell. Raw UMIs in each cell were both scaled and normalized via the SCTransform algorithm, regressing out unwanted source of variation arising from the percentage of mitochondrial genes. Genes with the most variable expression were identified and selected for principal component analysis (PCA) reduction of high-dimensional data. To reduce dimensionality, the RunUMAP, FindNeighbors, and FindClusters functions were used including the top 15 principal components. Gene expression levels were plotted with FeaturePlot and Dotplot functions.

### RNA extraction and NanoString nCounter assay

Total RNA was extracted from colonic tissues using the RNeasy kit (Qiagen) following the manufacturer’s instructions and quantified by NanoDrop. A total of 25 ng of RNA was used for NanoString nCounter assay, and the codeset for Mouse Fibrosis Panel (NanoString) was utilized (Table S2). The hybridization, processing, and acquisition were performed at the NanoString facility (NanoString Technologies). The normalization and differential expression analysis were conducted using NSolver 4.0 software (NanoString).

### Statistical analysis and software

All statistical analyses were performed in R (v. 4.3.1) and GraphPad Prism 10 (GraphPad Software, Inc.), and visualized with ggplot2 and Prism. Data are shown as the mean ± SEM. One-way or two-way analysis of variance followed by post hoc tests was used to compare different experimental groups, based on the factors defining the different comparisons, as indicated. The Mann–Whitney U test was used to compare two groups. Differences between groups were considered significant at a P value of <0.05. The graphical abstract was created in BioRender. Cortez (2025) https://BioRender.com/g8jitn7.

### Online supplemental material

Fig. S1 shows the characterization of the colonic and splenic immune responses of natural SSP and non-SSP hosts at 28 dpi. Fig. S2 provides additional data describing the colonic and splenic immune responses of F1-SSP (129X1/SvJ × C57BL/6) and ΔTreg SSP mice at 28 dpi. Fig. S3 presents additional data related to the scRNA-seq analysis of colonic Tregs isolated from F1-SSP and uninfected hosts, and to the characterization of colonic Tregs in SSP and non-SSP mice at 28 dpi. Fig. S4 presents additional data related to the scRNA-seq analysis of colonic Foxp3^^−^^ CD4^+^ T cells isolated from F1-SSP and uninfected hosts at 28 dpi. Fig. S5 shows that the depletion of either CD8^+^ T cells or the pro-inflammatory cytokine IFN-γ does not affect morbidity and mortality in ΔTreg SSP mice. Table S1 provides the grading scheme utilized to determine pathology scores. Table S2 provides the list of markers analyzed in the NanoString panel.

## Supplementary Material

Table S1 shows grading scheme for pathology scores.

Table S2 shows coverage across stages of the Fibrosis NanoString panel.

## Data Availability

All data generated in this study are presented in the manuscript and/or supplementary information. Any further information required for replicating experimental procedures will be made available by the corresponding authors upon reasonable request. The scRNA-seq data presented in Figs. 3 and 4 are generated in this study and are deposited in the GEO repository under the accession number GSE303864. The NanoString nCounter data presented in Fig. 5 are generated in this study and are deposited in the GEO repository under the accession number GSE302695.
