# Supplementary material for: Salmonella-superspreader hosts require gut regulatory T cells to maintain a disease-tolerant state
Source: J Exp Med. 2025 Sep 9;222(11):e20242431. doi: 10.1084/jem.20242431 (PMC12419162; doi:10.1084/jem.20242431)
Supplement: Table S1 — shows grading scheme for pathology scores. [file jem_20242431_tables1.docx]

**Table S1.** Grading scheme for pathology scores.

| **Criterion** | **Score** |
| --- | --- |
| **Ulceration** | 0= no ulceration  1= 1-5% of the surface area evaluated is ulcerated  2= 5-30% of the surface area evaluated is ulcerated  3= 30-70% of the surface area evaluated ulcerated  4= over 70% of the surface area evaluated is ulcerated |
| **Inflammation** | 0= no inflammation  1= small, focal, or widely separated areas of inflammation; often limited to the lamina propria  2= multifocal or coalescing areas of inflammation; can extend into the submucosa; minimal reduction in the density of crypts; minimal separation of crypt profiles  3= regionally extensive areas of inflammation; often extends to the submucosa (rarely transmural); multifocal paucity of crypt profiles; mild separation of crypt profiles  4= large regionally extensive to diffuse areas of inflammation; can be transmural; moderate paucity of crypt profiles; moderate separation of crypt profiles  5= diffuse inflammation; can be transmural; marked to severe paucity of crypt profiles; marked separation of crypt profiles |
| **Area involved/affected by inflammation** | 0= no inflammation  1= 1-5% involvement  2= 5-30% involvement  3= 30-70% involvement  4= over 70% involvement |
| **Edema and/or fibrin exudation** | 0= no edema or fibrin exudation  1= 1-5% involvement  2= 5-30% involvement  3= 30-70% involvement  4= over 70% involvement |
