# Supplementary material for: Salmonella-superspreader hosts require gut regulatory T cells to maintain a disease-tolerant state
Source: J Exp Med. 2025 Sep 9;222(11):e20242431. doi: 10.1084/jem.20242431 (PMC12419162; doi:10.1084/jem.20242431)
Supplement: Table S2 — shows coverage across stages of the Fibrosis NanoString panel. [file jem_20242431_tables2.docx]

**Table S2.** Coverage across stages of Fibrosis NanoString panel.

| **Stage** | **Description** | **Pathways** | **Number of Mouse Genes** |
| --- | --- | --- | --- |
| Initiation | Cell and tissue damage, often specific to an organ or fibrotic disease, initiates a cascade of stress and immune responses. | Autophagy, Cholesterol Metabolism, Cytosolic DNA Sensing, De Novo Lipogenesis, Endotoxin Response, Fatty Acid Metabolism, Gluconeogenesis, Insulin Resistance/Signaling, MAPK Cell Stress, mTOR, Oxidative Stress, PPAR Signaling, Proteotoxic Stress, SASP | 369 |
| Inflammation | Inflammation is one of many responses to the initial damage, involving multiple immune cell types and signaling pathways. Chronic inflammation drives the proliferation of pro-fibrotic cells and tissue modification. | Adenosine Pathway, Chemokine Signaling, Complement Activation, Cytokine Signaling, Granulocyte Activity, Inflammasome, M1/M2 Activation, MHC Class II Antigen Presentation, Neutrophil Degranulation, NF-kB, Phagocytic Cell Function, Platelet Degranulation, Th1/ Th2/Th17 Differentiation, TLR Signaling, Type I/Type II Interferon | 297 |
| Proliferation | Differentiation and proliferation of myofibroblasts are driven by upstream inflammation. These cells drive the wound-healing response that results in fibrotic damage. | Cell Cycle, ECM Synthesis, EMT, Focal Adhesion Kinase, Hedgehog Signaling, Hypoxia, Myofibroblast Regulation, Notch, PDGF Signaling, PI3K-Akt, Tgf- Beta, Wnt | 161 |
| Modification | Immune and myofibroblast cells contribute to extracellular matrix modification and tissue alterations that are characteristic of fibrotic disease. | Angiogenesis, Apoptosis, Collagen Biosynthesis & Modification, ECM Degradation, Epigenetic Modification, Hippo Pathway, Regulated Necrosis | 287 |

Adapted from: https://www.nanostring.com/products/ncounter-assays-panels/immunology/fibrosis
